# Hotspots of Dissolved Arsenic Generated from Buried Silt Layers along Fluctuating Rivers

**DOI:** 10.1021/acs.est.4c02330

**Published:** 2024-08-13

**Authors:** Kyungwon Kwak, Thomas S. Varner, William Nguyen, Harshad V. Kulkarni, Reid Buskirk, Yibin Huang, Abu Saeed, Alamgir Hosain, Jacqueline Aitkenhead-Peterson, Kazi M. Ahmed, Syed Humayun Akhter, M. Bayani Cardenas, Saugata Datta, Peter S. K. Knappett

**Affiliations:** † Department of Geology and Geophysics, Texas A&M University, College Station, Texas 77843, United States; ‡ Department of Earth and Planetary Sciences, The University of Texas at San Antonio, San Antonio, Texas 78249, United States; § Department of Earth and Planetary Sciences, The University of Texas at Austin, Austin, Texas 78712, United States; ∥ Department of Geology, University of Dhaka, Dhaka 1000, Bangladesh; ⊥ Department of Coastal Studies and Disaster Management, University of Barishal, Barishal 8200, Bangladesh; # Department of Soil & Crop Science, Texas A&M University, College Station, Texas 77845, United States; ∇ School of Civil & Environmental Engineering, Indian Institute of Technology Mandi, Himachal Pradesh 175075, India

**Keywords:** arsenic (As), natural reactive barrier (NRB), surficial sediment, surface water-groundwater interactions, hyporheic zone, tidal fluctuations, monsoonal river fluctuations

## Abstract

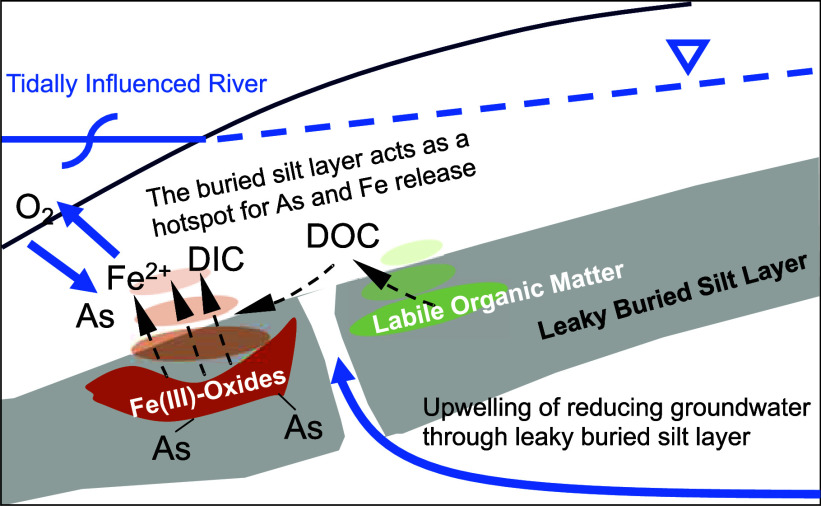

Previous studies along the banks of the tidal Meghna River of the Ganges-Brahmaputra-Meghna Delta demonstrated the active sequestration of dissolved arsenic (As) on newly formed iron oxide minerals (Fe(III)-oxides) within riverbank sands. The sand with high solid-phase As (>500 mg/kg) was located within the intertidal zone where robust mixing occurs with oxygen-rich river water. Here we present new evidence that upwelling groundwater through a buried silt layer generates the dissolved products of reductive dissolution of Fe(III)-oxides, including As, while mobilization of DOC by upwelling groundwater prevents their reconstitution in the intertidal zone by lowering the redox state. A three end-member conservative mixing model demonstrated mixing between riverbank groundwater above the silt layer, upwelling groundwater through the silt layer, and river water. An electrochemical mass balance model confirmed that Fe(III)-oxides were the primary electron acceptor driving the oxidation of DOC sourced from sediment organic carbon in the silt. Thus, the presence of an intercalating silt layer in the riverbanks of tidal rivers can represent a biogeochemical hotspot of As release while preventing its retention in the hyporheic zone.

## Abstract 2

We identified a new type of riverbank with respect to the source and fate of dissolved arsenic. Buried silt layers along tidally influenced river corridors create biogeochemical hotspots that release dissolved As and prevent its retention in the hyporheic zone.

## Introduction

Arsenic (As) contamination of groundwater in Bangladesh continues to be the largest case of human poisoning in history.^1,2^ One important component of the multipronged strategy to mitigate human exposure to As in drinking water is to unravel the fundamental hydrological and geochemical mechanisms that drive the heterogeneous distribution of As concentrations in South and Southeast Asia.^3−8^ Freshly deposited, fluvial sediments have recently been noted for their role in supplying fresh organic carbon (OC) that drives microbially mediated reductive dissolution of iron oxide minerals (Fe(III)-oxides), thereby releasing dissolved As and iron (Fe(II)) to adjacent aquifers.^8−14^ In the riverbank hyporheic zone (HZ), regular tidal or episodic river stage fluctuations regulate the redox state of the HZ^15^ and enhance transport of nutrients and contaminants.^16,17^ The transport of the oxidants dissolved oxygen (DO) and nitrate (NO_3_^–^) from river water into reducing aquifers generates biogeochemical hot spots.^18^ In reducing riverbanks below 0.5 m depth along the low energy Meghna River, DO and NO_3_^–^ are scarce.^19^ In such settings mixing with river water may nonetheless generate dissolved As hotspots by the colocation of amorphous solid-phase Fe(III)-oxides, with three OC sources: sediment organic carbon (SOC) from seasonal deposition and tidal reworking along the intertidal zone riverbank; recalcitrant, but abundant dissolved organic carbon (DOC) advected toward the river from the aquifer, and labile river DOC.^8,9,11−14^ These reactants mix under the influence of semidiurnal tides.

**Figure 1:**
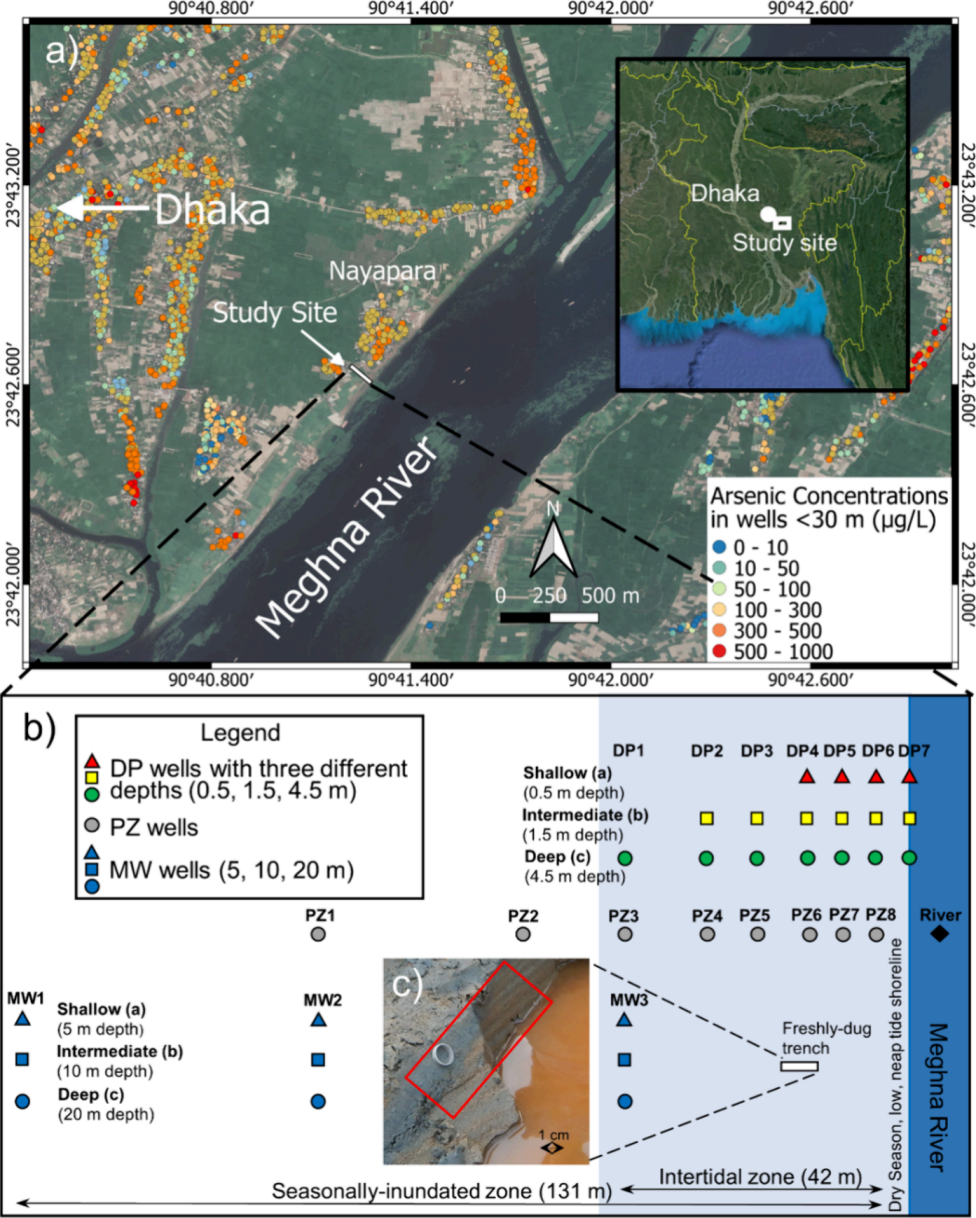
Location of the study site and configuration of sampling wells. (a) Location of the study site on the western bank of the Meghna River. Dissolved As concentrations were measured in private wells during 2012–2013 reproduced as reported by van Geen et al.^31^ (b) Across the 131 m-wide transect oriented orthogonally to the river shoreline, three types of wells were installed: (i) Drive-point piezometers (DP): “DPa” wells (∼0.5 m), “DPb” wells (∼1.5 m), and “DPc” wells (3 to 4.5 m); (ii) Fully screened shallow piezometers (PZ); (iii) Monitoring wells (MW): “MWa” wells (∼5 m), “MWb” wells (∼10 m), and “MWc” wells (∼20 m). All wells were numbered in descending order away from the river. For example, the DP well that is furthest from the river and has the shallowest depth is referred to as “DP1a”. (c) Freshly dug trench within the intertidal zone oriented orthogonally to the Meghna River shoreline. Alternating laminations of gray and orange layers in the sediment represent predominantly Fe-reducing and Fe-oxidizing conditions, respectively. Picture taken at Nayapara study site on Jan. 5, 2020.

Whether As accumulates in sediments within the HZ, or is released to porewaters, is regulated by the redox state which is determined by the chemical stability (recalcitrance) of OC and Fe(III)-oxides.^19−23^ Receding monsoonal flood waters annually replenish riverbank sediments. In the dry season, semidiurnal and neap-spring tides drive frequent, robust mixing of oxidizing river water and reducing groundwater across the freshly deposited riverbank sediments in parafluvial zones.^15,24,25^ At our study site (Figure 1), thin (∼1 cm) laminations of alternating gray and orange layers were observed within the surficial deposits up to approximately 0.5 m depth (Figure 1c). The gray and orange layers represent sediments that contain predominantly reduced (Fe(II)) and oxidized (Fe(III)) iron, respectively.^26^ Parafluvial and river bed sediments along the Red, Mekong, and Meghna Rivers contain high concentrations of adsorbed As, reactive Fe(III)-oxides, and fresh labile OC.^8,9,12,27−29^ These sediments host the dynamic river-aquifer interface in which reducing groundwater and oxidizing river water mix. Reducing and oxidizing conditions favor the dissolution and precipitation of Fe(III)-oxides, respectively. Therefore, the redox state of the sediments and porewaters in this zone may play a critical role in mobilizing or immobilizing dissolved As and Fe.

Seasonally inundated parafluvial zones along tidally fluctuating rivers in Asia host countless communities who rely on groundwater as their primary source of freshwater (Figure 1a).^1,2,8,30,31^ However, the impact of transient inundation and variably saturated surficial sediments across the tidally driven mixing zone on the mass fluxes of As and Fe(II) toward the river has been sparingly studied. Of the evidence published to date, there is broad consensus that permeable riverbank aquifers accumulate the mass fluxes of As discharging to rivers.^19,23,27,28,32^ Laboratory experiments demonstrated that repetitive cycling between oxidizing and reducing conditions immobilizes dissolved As within the HZ.^21^ This laboratory finding is consistent with the results of recent field studies, in which dissolved As was observed to actively accumulate in the HZ under bidirectional mixing along the tidal Meghna River.^19,22,23^ Huang et al.^22^ demonstrated that the mass fluxes of dissolved As advecting from the adjacent shallow Holocene alluvial aquifers are sufficient to account for the mass of sedimentary As accumulated in the riverbank sediment at four separate sites along the Meghna River. Thus, dissolved As generally does accumulate within bidirectional mixing zones within the banks of tidally fluctuating Meghna River. However, the fate of dissolved As in groundwater discharging to the river and its behavior within these parafluvial zones are not yet fully understood. It is important to understand the fate of many kilograms of dissolved As discharging to rivers^22^ to comprehend how As is cycled between river sediment, floodplains, and groundwater in South and Southeast Asia. Depending on the regional hydrology, surficial lithology, and geochemical settings, these parafluvial zones can either serve as a sink for the As flux discharging to the river^19,23,28,32^ or act as a source adding new dissolved As into the broader shallow aquifer.^12^ This study contributes to understanding how As cycling is influenced by riverine hydrology, floodplain geomorphology and geochemistry, and groundwater flow and transport processes.

## Materials and Methods

### Study Site

The study site is located on the western bank of the Meghna River, adjacent to a village called Nayapara (New Village) in Araihazar upazilla (subdistrict) (see details in Text S1). Araihazar is located in central Bangladesh approximately 30 km east of Dhaka (Figure 1a).

### Aquifer Properties

To identify sandy riverbanks and resolve the dimensions of the aquifer and underlying silt and clay layers, electrical resistivity imaging (ERI) was utilized (Figure S1).^33^ Once the site was selected borehole lithologies were obtained from drill cuttings using the local reverse circulation hand-flapper method.^34^ The borehole lithologies and the ERI measurements constrained a detailed 2D geological model of the site (see details in Text S2).^33^ From top to bottom, the geology of the site is comprised of six hydrogeologic units: (i) an approximately 2 m thick fine-sand vadose zone that is represented in the ERI model as a high resistivity zone (>180 Ωm); (ii) an approximately 3 m-thick fine-sand zone, herein referred to as the riverbank aquifer, which thins toward the river (100 to 140 Ωm); (iii) a 4 to 5 m-thick leaky silt aquitard, herein referred to as the buried silt layer (40 to 60 Ωm); (iv) a 12 m thick medium to coarse-sand aquifer (140 Ωm), herein referred to as the shallow Holocene aquifer; (v) a 7 m medium-sand layer (∼100 Ωm); and vi) a regional clay aquitard at 27 m depth (Figure S1). The two borehole lithologies (BH1 and BH2) agreed closely with the ERI results (Figure S1).^33^ Generally, the sediment below the dry season water table was gray in color down to the underlying clay aquitard. The generally high dissolved As concentrations found in the gray sand within the riverbank aquifer at this site are consistent with other studies that find that gray sand is associated with high As concentrations in shallow aquifers across the Ganges-Brahmaputra delta.^5,26^ The buried silt layer is a leaky aquitard and discontinuous. The entire 131 m wide riverbank is inundated each wet season from early July to September. The majority of annual rain (2076 mm) falls during the monsoon season (June to October).^35^ The water table peaks approximately when the river peaks, but it declines more gradually reaching its nadir by the late dry season (March to April).^19,36−38^ This drives strong gaining conditions for the river during the early dry season.

### Well Installation, Hydraulic Testing, and Water Level Monitoring

A total of 37 monitoring wells and drive-point piezometers were installed across a 131 m transect oriented orthogonally to the river which spans the range of the river shoreline throughout the year (Figure 1b). The transect is composed of three different types of wells to achieve different aims: (i) 17 drive-point piezometers, hereafter referred to as the DP wells, were installed to collect depth-specific porewater samples to analyze for chemical and isotopic composition in the riverbank aquifer above and within the buried silt layer and across the breadth of the neap-spring intertidal zone to depth at 0.5 m (“DPa” wells), 1.5 m (“DPb” wells), and 4.5 m (“DPc” wells); (ii) 9 monitoring wells, hereafter referred to as the MW wells, were installed to observe hydraulic heads and depth-specific porewater chemistry across the breadth of the riverbank that spans the seasonally inundated zone. The positions of the screens of the MW wells targeted the range of dry season water table fluctuations (“MWa” wells; ∼ 5 m depth), just below the buried silt layer (“MWb” wells; ∼ 10 m depth), and near the bottom of the lower shallow Holocene aquifer (“MWc” wells; ∼ 20 m depth); and last (iii) 8 piezometers were augured in with screen intervals that spanned the vertical range of tidally driven water table fluctuations that occurs during the dry season. These are referred to hereafter as the PZ wells. All DP, MW, and PZ wells were numbered in descending order away from the river. For example, the DP well that is furthest from the river and has the shallowest depth is referred to as “DP1a”.

The DP wells were composed of a stainless-steel drive-point piezometer head with 15 cm screen (Model 615, Solinst Canada Ltd.) and a 16 mm outer diameter stainless-steel pipe. Vertical nests of DP wells were installed at seven locations. These wells were installed across the dry season, neap-spring (14 days) intertidal zone of the riverbank, which spans a distance of approximately 42 m (Figure 1b).

The MW wells were composed of 5.08 cm (2 in.) inner diameter PVC casing with 1.5 m long screens. The local “hand-flapper” method was used to drill boreholes before installing the PVC pipes.^34^ Three vertical nests of three wells each were installed within separate boreholes. Whereas the 10 m-deep MWb and the 20 m-deep MWc wells had 1.5 m screens, the 5 m-deep MWa wells were continuously screened to observe the movement of the water table during all seasons and to sample the porewater composition of the water table during the dry season. The three MW nests were located at distances spanning from 42 to 131 m inland, from the low, neap tide shoreline in the dry season. The peak monsoon shoreline at 131 m from the dry season shoreline is a built-up roadway that connects two parts of the village. During the midmonsoon, the river inundates large rice fields to the west of this roadway. Thus, the aquifer is recharged by early monsoon rainfall (May to June) and midmonsoon riverine floodplain recharge (July).^39^ The estimated average annual groundwater recharge in the Dhaka area is ∼2065 mm/year per unit area.^40^ Once the groundwater table has been recharged and raised to the ground surface, little additional recharge occurs.^38^ Thus, the principal recharge water is a mixture between the early monsoon rainfall and midmonsoon river water. The MWb and MWc wells sample the groundwater chemistry of the shallow Holocene aquifer below the buried silt layer. The MW well nests were spaced approximately 45 m apart laterally (Figure 1b). After installation, all DP wells and MW wells were purged for 10 min prior to sampling to flush stagnant water.

The PZ wells were composed of 3.18 cm (1.25 in.) inner diameter, slotted PVC pipes. These wells were installed to monitor the water table and were continuously screened.

### Aqueous Chemistry Analysis

Water samples were collected along the transect (Figure 1b) between January 11th and 14th, 2020. The DP and PZ wells were pumped with a peristaltic pump (Model 410, Solinst Canada Ltd.) with a flow rate of approximately 50 mL/min. The MW wells were pumped using a plastic submersible pump (Typhoon Model, Groundwater Essentials LLC.) with a flow rate of approximately 2–4 L/min. Parameters including temperature, pH, specific conductance (SC) and Oxidative–Reductive Potential (ORP) were measured using a multimeter sensor which was calibrated daily (YSI Professional Plus, YSI Inc.). Prior to sampling, each well was purged and pumped until temperature, pH, SC, and ORP stabilized. Redox-sensitive parameters including DO, NO_3_^–^, ammonium (NH_4_^+^), sulfide (H_2_S), manganese (Mn(II)), and Fe^(^II) were measured on-site using colorimetric tests with a portable spectrophotometer (V-2000, CHEMetrics Inc.). Approximate concentrations of dissolved total As were measured on-site using a colorimetric arsenic test kit (Econo-Quick arsenic test kit, Industrial Test Systems Inc.). Alkalinity was measured by titration with 1 M H_2_SO_4_ and methyl red bromocresol green pH 5 indicator (Model AL-DT alkalinity test kit, HACH Company).

All water samples for laboratory measurements were filtered on-site through a 0.45 μm nitrocellulose syringe filter (Millipore Millex – HP, Merck KGaA) into acid-cleaned 20 mL High Density Polyethylene (HDPE) vials. Water samples for major cations (Ca^2+^, K^+^, Na^+^, Mg^2+^) and the redox-sensitive dissolved elements (Fe, Mn, and As) were acidified with Optima grade HNO_3_ (2% v/v) and analyzed using inductively coupled plasma spectroscopy (ICP-MS) (Element XR, Thermo Scientific). Water samples for As speciation measurements were filtered through an arsenic speciation cartridge (Arsenic Speciation Cartridge, MetalSoft) to separate As(III) from total As. Water samples for stable water isotope (δ^18^O and δD) were filtered, stored in 20 mL clear glass vials, and analyzed by a cavity ring-down instrument (Picarro L2120-i CRDS, Picarro Inc.). Water samples for DOC analysis were filtered through 0.7 μm diameter ashed GF/F syringe filters (Whatman, Cytiva) and stored in 40 mL amber glass vials that were preloaded with 20 μL of 12 M Optima grade HCl. The DOC samples were then analyzed as nonpurgeable organic carbon (NPOC) by a total organic carbon analyzer (TOC-VCSH, Shimandzu).

Detailed descriptions of methods for measuring aqueous chemistry both in situ and in the laboratory can be found in the Supporting Information (Text S1).

### Three End-Member Mixing Model

To evaluate the proportional contribution of distinct water sources within the riverbank, δ^2^H, δ^18^O, and chloride (Cl^–^) were utilized as conservative tracers. Little evaporation effects were observed such that δ^2^H and δ^18^O plotted directly on the Local Meteoric Water Line (LMWL) (Figure S8, Text S4). Therefore, Cl^–^ concentration and δ^18^O were utilized as conservative tracers to constrain a three end-member mixing model (Text S4). The following three end-members were used to describe the composition of these conservative tracers in porewaters in the shallow intertidal zone: (i) riverbank groundwater; (ii) shallow Holocene groundwater; and (iii) river water. The riverbank groundwater end-member is positioned above the buried silt layer (MW1a). The shallow Holocene groundwater end-member is positioned below the buried silt layer. The early monsoon river water was used to represent the shallow Holocene groundwater end-member because their δ^18^O and Cl^–^ compositions are similar. This is because the shallow aquifer is recharged during the early monsoon by local rainfall and riverine flooding as local and regional studies have demonstrated.^39^ A river water sample taken at the time of this study (January, dry season) represents the river water end-member that is actively mixing with groundwater in the riverbank. The relative contributions of individual water sources were quantified with a ternary mixing model. Detailed descriptions for the mixing model can be found in the Supporting Information (Text S4).

### Electron Transfer Calculations

The number of electrons transferred during the oxidation/mineralization of DOC was calculated to constrain the redox reactions, which drive the in situ release of dissolved As and Fe into porewaters. Specifically, the mmol of electron donors (ED) oxidized and electron acceptors (EA) reduced in both the aqueous phase and the sediments were calculated.^41^ This calculation assumes that the oxidation of OC is the primary source of DIC and that there are no significant sinks of DIC. In the calculation, the major ED was DOC and the major EA’s considered were Fe(III), Mn(IV), SO_4_, and O_2_. The calculation was performed using the equation below.^41^
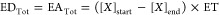
1where ED_Tot_ and EA_Tot_ are the milliequivalents (meq/L) of all electrons donated and accepted in the aqueous phase along the flow paths, respectively. The terms [*X*]_start_ and [*X*]_end_ are the molar concentrations (mmol) of redox-sensitive chemical species at the start and end of the flow path. Lastly, *ET* is the moles of electrons transferred per mole of OC oxidized. The justification for the equality of ED_Tot_ and EA_Tot_ is that each electron transferred during DOC oxidation must have been received by an available EA. This model implicitly assumes flow of a plug of groundwater along the same stream tube that is sampled by both the upstream and downstream well. It does not explicitly account for mixing with river water or upwelling groundwater. Electrons transferred for three different pairs of start and end points of flow paths were calculated (see Figure S2 for the three different pairs).

## Results and Discussion

### Mixing between three water end-members in the banks

**Figure 2:**
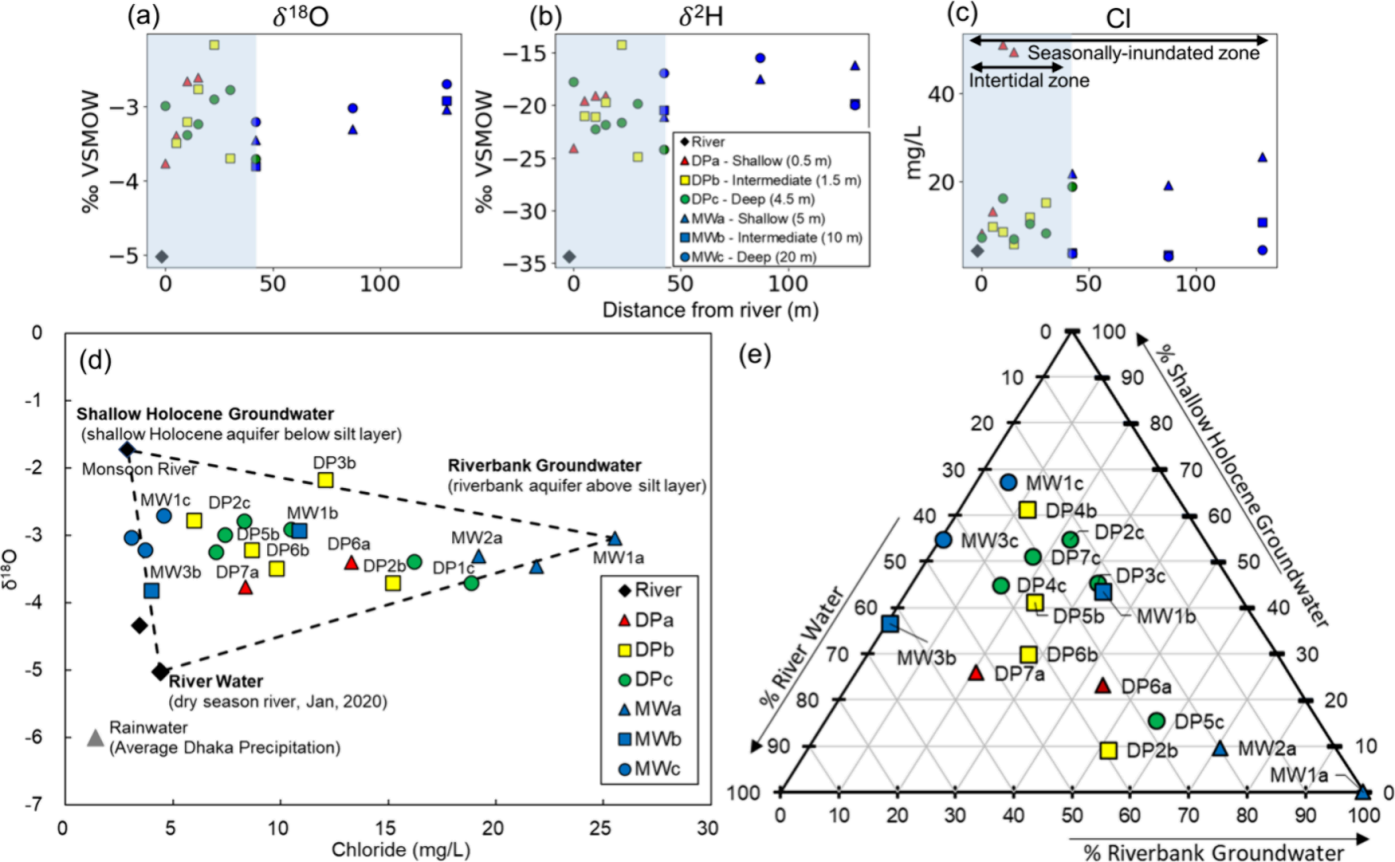
Spatial distribution of conservative tracers and three end-member mixing model. (a-c) Spatial distribution of conservative tracers (δ^18^O, δ^2^H, and Cl^–^). The decreasing trend of three conservative tracers indicates mixing between groundwater and river water. Red triangle, yellow square, and green circles represent the DP well samples where they are installed in the shallow intertidal zone (0 to 50 m far from the river and 0.5 to 5 m deep). Blue triangles, squares, and circles represent MWa (∼5 m), MWb (∼10 m), and MWc (∼20 m) wells. The black diamond represents river water. The *x* axis describes the lateral distance of each well from the dry season river shoreline during low, neap tide. (d) Three end-member mixing model. The end-members are riverbank groundwater, shallow Holocene groundwater, and river water. The mixing model described approximately 90% of DP and MW well samples and required three end-members, suggesting that there is mixing between the river water and groundwater from both above and below the silt layer. (e) Ternary diagram showing the relative proportions of three end-members in each well.

Flow paths were inferred from the stratigraphy (Figure S1), observed lateral and vertical hydraulic gradients (Figure S5), spatial patterns in the conservative tracers (δ^18^O, δ^2^H, and Cl^–^) (Figure 2a,b,c), and the formal mixing calculations (Text S4).^19^ Two distinct groundwater flow paths were identified that generally flow toward the river. One flows within the shallow intertidal zone above the buried silt layer (0 to ∼5 m) (DP wells and MWa wells), and another flows within the shallow Holocene aquifer (>10 m) (MWb and MWc wells) and upwells through the buried silt layer (Figure S1). Both δ^18^O and Cl^–^ concentrations decreased toward the river along the shallow intertidal zone (∼-2 to ∼ −4 ‰ and 20 to 3 mg/L, respectively) (Figure 2a,c). This suggests that there is a mixing between the river water and the riverbank groundwater in the intertidal zone. The vertical hydraulic gradients in the DP wells indicated that deeper groundwater from the shallow Holocene aquifer upwells into the intertidal zone (Figure S5).

Although hydraulic gradients indicate that upward flow is occurring in the intertidal zone, they are insufficient to determine whether this is a negligible or substantial volumetric flux. To determine this, we quantified mixing between river water and groundwater from above and below the buried silt layer using δ^18^O and Cl^–^ as conservative tracers (Figure 2d,e, Figure S10, Figure S11, Table S2, Table S3). The three end-member mixing model could explain the composition of approximately 90% of all wells screened within the intertidal zone (Figure 2d, Figure S10). Many DP wells installed within and above the buried silt layer contained high proportions of groundwater from below the silt layer (63% to 95%). The results support the hypothesis that there is mixing between all three end-member waters above and below the silt layer and agree with the vertical hydraulic gradients (Figure S5, Table S2, Table S3). Thus, groundwater flow paths converge within and above the buried silt layer at the river’s edge (Figure S1). This is consistent with theoretical and conceptual models of groundwater discharge to surface water bodies.^42−46^

### Dissolved As and Fe increased toward the river

**Figure 3:**
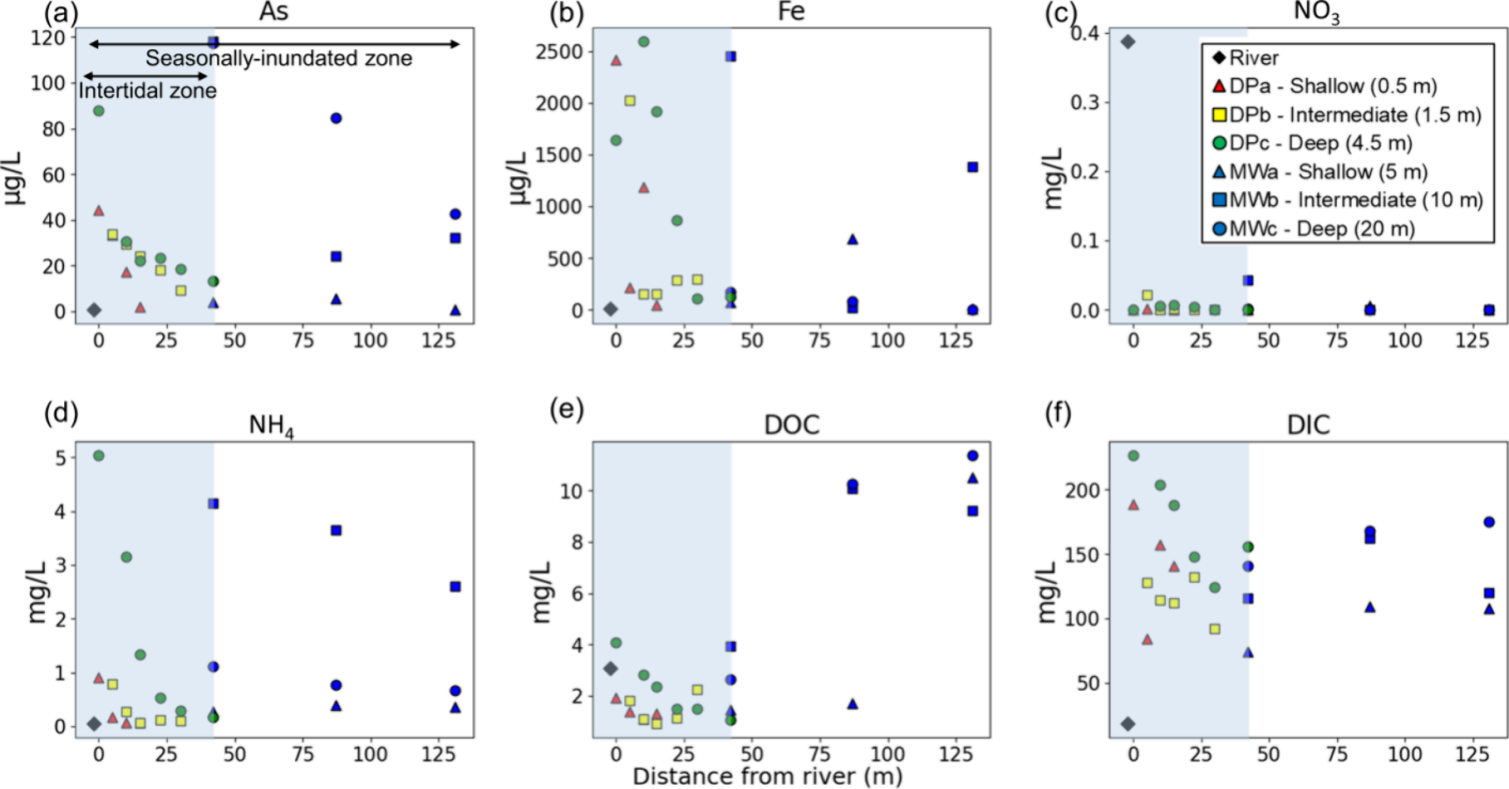
Porewater chemistry profile across the intertidal zone (blue box) and the seasonally inundated zone across the riverbank. Redox-sensitive elements As, Fe(II), NO_3_ and NH_4_ (a-d) and dissolved carbon species (e-f). Red triangles, yellow squares, and green circles represent DPa (∼0.5 m), DPb (∼1.5 m), and DPc (∼3 to 4.5 m) wells, respectively. Blue triangles, squares, and circles represent MWa (∼5 m), MWb (∼10 m), and MWc (∼20 m) wells, respectively. The black diamond represents dry season river water composition. The *x* axis describes the distance of each well from the dry season river shoreline during low, neap tide.

From the water table down to 4.5 m below the riverbank surface, the dissolved concentrations of the products of the reductive dissolution of Fe(III)-oxides, including As, Fe(II), DIC, and NH_4_^+^, remained low across the riverbank until the flow paths entered the intertidal zone (Figure 3a,b,d,f). Within this shallow intertidal zone above the buried silt layer (DP wells) (Figure 1b, Figure S1), their concentrations rapidly increased toward the river (Figure 3a,b,d,f) concomitant with an increase in the magnitude of vertical hydraulic gradients favoring upward flow. Across the riverbank, dissolved As concentrations increased from 23 to 164 μg/L and Fe concentrations increased from 12 to 1858 μg/L (Figure 3a,b). The river contained relatively low dissolved As and Fe(II) concentrations (4 μg/L and 2 μg/L). Along the same flow path, DOC concentrations increased from 1 to 4 mg/L (Figure 3e) whereas concentrations of dissolved inorganic carbon (DIC), measured as bicarbonate (HCO_3_^–^), increased from 92 to 227 mg/L (Figure 3f). Concentrations of NH_4_^+^ increased toward the river (0.06 to 5.04 mg/L) (Figure 3d). Concentrations of DO and NO_3_^–^ within all sampling points were negligible (<1 mg/L and <0.1 mg/L, respectively) (see Figure 3c and Figure S6e for additional chemical data). This pattern of increasing As and Fe concentrations can be explained by the microbially mediated reductive dissolution of Fe(III)-oxides by oxidation (heterotrophic respiration) of DOC along the final ∼40 m of transport path toward the river along the shallow flow path above the buried silt layer.

At a previously characterized nearby study site composed of uniform sand, at the same time of year, dissolved Fe(II) was released to porewaters across the shallow intertidal zone flow path whereas dissolved As concentrations remained low.^19^ This finding from this previous study implies that in riverbanks with high permeability uniform sand, even as Fe(III)-oxides are dissolved during the early dry season, sufficient Fe(III)-oxides remain to resorb dissolved As advecting to the river from the aquifer.^19^ Results from a preliminary reactive flow and transport model of that sandy site suggested that permeable isotropic riverbanks regenerate their Fe(III)-oxides within the intertidal zone during dry season conditions.^17,47^ However, at the new study site we present here, the reduction of the pool of Fe(III)-oxide within the bidirectional mixing zone is more extensive than at the previously characterized sandy site as evidenced by the increase in dissolved Fe(II) and As concentrations (Figure 3, and see Figure S1 for the hydro-stratigraphy of the site).

### Source of fresh labile organic matter

The DOC that river water carries may be an important ED source for indigenous bacteria within the HZ.^48,49^ However, the largest mass of OC that may drive the observed reductive dissolution is the DOC sourced from SOC within the buried silt layer. The buried silt layer in the riverbank (Figure S1) is the primary source of both DOC and As in the shallow intertidal zone. It is widely recognized that DOC diffuses, or under falling pore pressures from groundwater pumping, is expulsed from silt and clay layers into adjacent aquifers. This DOC may be accompanied by dissolved Fe(II) and As, and the DOC may go on to drive reductive dissolution of Fe(III)-oxides and release more dissolved As from the adjacent aquifer sands.^50−53^ At our site, the evidence supports the upwelling of groundwater through the buried silt layer mobilizing DOC from the silt into the overlying sand layer in which robust mixing with the river occurs (Figure S5). Although others have shown that silt and clay compaction expulse As and DOC,^51−53^ to the authors’ knowledge it has not previously been demonstrated that the upwelling of deeper groundwater mobilizes DOC from silt. The expulsion of DOC from silts and clays has usually been associated with depressurization within aquitards from aquifer pumping. At our site, we show that locally produced DOC from the silt layer drives the reductive dissolution of Fe(III)-oxides within the shallow intertidal zone, and then goes on to lower the redox state in the shallow sand thereby preventing new Fe(III)-oxides from forming. The active decomposition of DOC/SOC is further supported by the fact that the majority of the groundwater samples (DP wells and MW wells) contained chloride (Cl^–^)/bromide (Br) mass ratios lower than 200 (Text S5, Figure S12). Such low ratios imply that natural organic matter is actively decomposed across the riverbank.^54−56^ Dissolved organic carbon expulsion from the buried silt layer is supported by the observed concomitant increase in DOC as well as decomposition products of OC like DIC and NH_4_^+^ within and above the silt layer across the intertidal zone (Figure 3d,e,f).

Electron transfer calculations provide quantitative evidence to test the hypothesis that Fe(III)-oxides in riverbank sediment are reacting with labile organic matter (Text S6, Figure S2, Table S4, Table S5, Table S6). These calculations reveal that the observed high DIC concentrations produced from OC oxidation cannot be explained by the available dissolved ED and EA existing in porewaters. Even though there is evidence of upwelling of deep groundwater along the riverbank within the intertidal zone, the observed DIC concentration far exceeded that in the shallow Holocene aquifer. Therefore, the DIC must be produced within these shallow intertidal flow paths within and above the buried silt layer. Since DO and NO_3_^–^ are nearly absent, and rising dissolved manganese (Mn(II)) and falling sulfate (SO_4_^2–^) concentrations are too little for Mn(IV)-oxides and sulfate to be the primary electron acceptors, Fe(III)-oxides and SOC must be the main EA and ED along the flow paths (Text S6, Table S4, Table S5, Table S6). The SOC donates electrons to Fe(III)-oxides. These EA-ED pairs may either be colocated or the decomposition of SOC generates mobile DOC which then becomes the ED when it comes into contact with stationary Fe(III)-oxides. Mixing with the river water within the buried silt and overlying sands may introduce small amounts of labile riverine DOC and enhance the rate of microbial respiration of more recalcitrant groundwater DOC or SOC.^48^ Also, the downward percolation of water through the freshly deposited overbank SOC during ebb tide may bring fresh DOC into the upper 0.5 m of the sand, which is the next most productive layer of As, Fe(II) and the other associated substance, after the buried silt. None of the three end-member waters contained sufficient DOC to account for the mass of DIC produced. Therefore, SOC had to be the main source of ED.

The strong role of the SOC in driving reductive dissolution of Fe(III)-oxides within the intertidal riverbank is consistent with findings from the complementary studies that we conducted with sediment from the three riverbank layers to measure the lability and availability of sedimentary organic matter.^14,57^ The leachates of shallow (∼1 to 5 m) riverbank sediment contained organic matter with a low molecular weight (0.1 kDa) and a low humic:protein ratio of 0.2. The silt layer, however, contained much higher concentrations of water-extractable organic matter (1274 mg/kg) compared to the sand above the silt layer (67 mg/kg).^14^ The bulk organic matter in the buried silt layer contained the highest proportions of bioavailable carbohydrate and carbonyl functional groups (26.1%) compared to the riverbank sand above the silt layer (17.5%) or the shallow Holocene aquifer sediment below the silt layer (12.1%).^14,57^ This indicates that the organic matter in the silt layer has the highest levels of microbial activity and highest potential to be utilized as an ED for heterotrophic microbial respiration compared to the sand layers above and below the silt layer. Together, these findings suggest that the riverbank sediment and the buried silt layer contain more young, labile sedimentary organic matter with higher electron donating capacities than the underlying shallow Holocene aquifer sediment. This labile SOC promotes the reductive dissolution of Fe(III)-oxides within the HZ.

### Buried silt layer: hotspot for As

**Figure 4:**
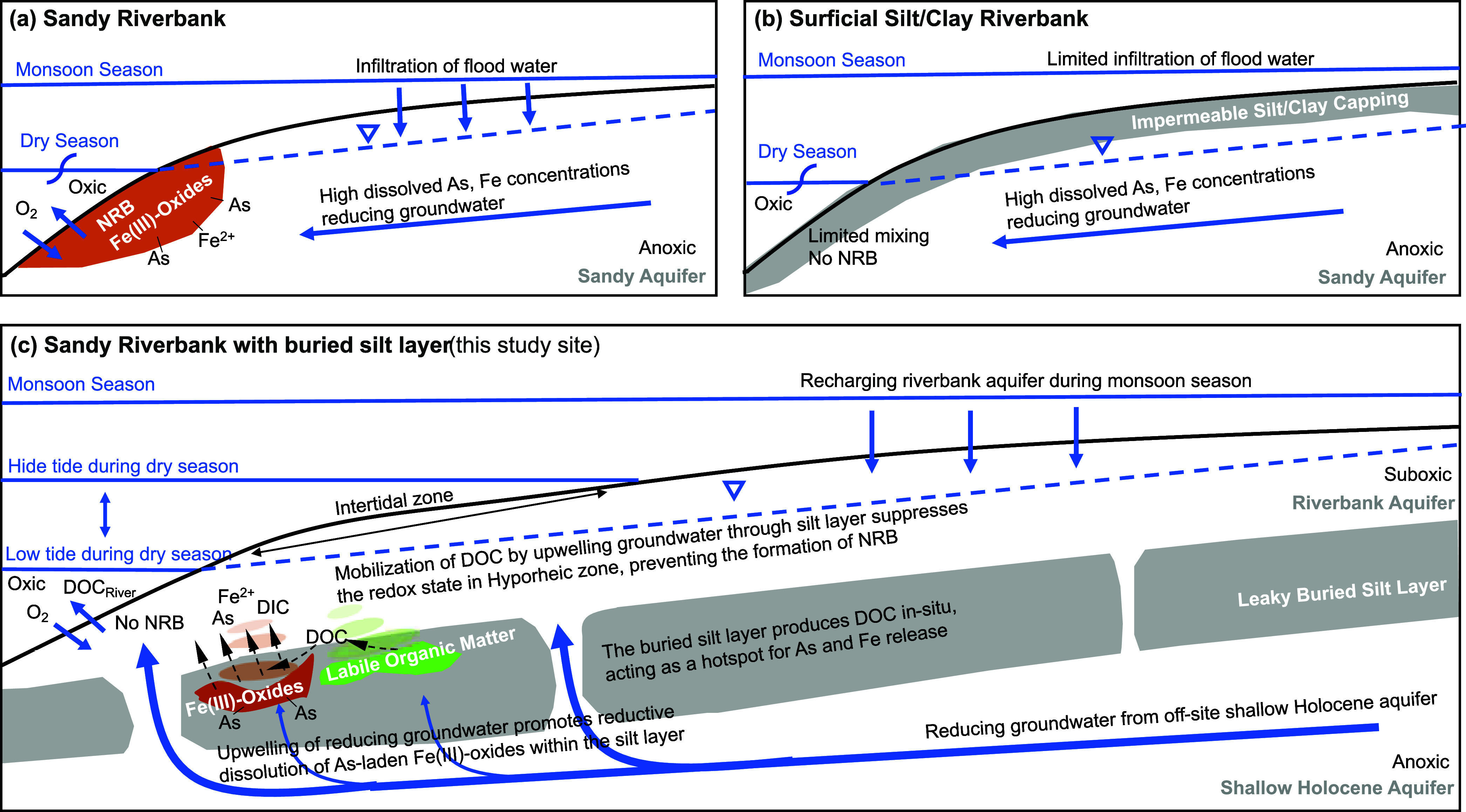
Schematic model of three possible scenarios at the end of the flow path of riverbank aquifer. (a) Formation of NRB along permeable sandy riverbank reproduced from Jung et al.^28^ and Berube et al.^19^. (b) No formation of NRB due to limited mixing between oxidizing surface water and groundwater.^28^ (c) Conceptual model of role of leaky buried silt layer in releasing As to groundwater at this study site. Mobilization of DOC by upwelling groundwater though the buried silt layer suppresses the redox state in HZ. The buried silt layer prevents As accumulation within the HZ on Fe(III)-oxides across intertidal zones, and instead generates a biogeochemical hotspot for As release within the HZ. The difference of river stages during monsoon season and dry season is approximately 4 m. The thin, dashed black lines represent elements that are chemically transformed along the way. The thick, blue lines represent groundwater flow paths. This diagram is not to scale. Approximate vertical exaggeration of this figure is 10.

The findings described above collectively show that under the influence of dry season tidal fluctuations, the buried silt layer is an active source of dissolved As. During mixing of surface water and groundwater driven by semidiurnal tides, this buried silt layer acts as a biogeochemical hotspot releasing As to the HZ through microbially mediated reductive dissolution of Fe(III)-oxides across the intertidal zone along the Meghna River (Figure 4 and see Table S7 for the key chemical reactions). Under reducing conditions, the SOC and DOC donate electrons to Fe(III)-oxides which release CO_2_ to the porewater (Figure 4 and Table S7). The released CO_2_ forms carbonic acid and that acidity is attenuated by chemically weathering carbonate or silicate minerals thereby contributing to the bicarbonate pool which was measured as DIC (Figure S7).^58^

Recent studies have suggested that in a sufficiently permeable riverbank a natural reactive barrier (NRB), which acts as a sink for dissolved As and Fe, forms 1–5 m below the river-aquifer interfaces (Figure 4a).^19,20,27,28,32,59,60^ However, the dissolved Fe(II) and As are not actively accumulating in the solid-phase at the present study site in spite of a permeable surficial sand and robust mixing between the dissolved Fe(II)-rich groundwater and the oxygen-rich river water. This lack of an NRB was confirmed by low solid-phase concentrations of Fe (40 ± 6 g/kg) and As (7 ± 1 mg/kg) which were measured with benchtop X-ray Fluorescence (XRF).^14^ This is because the buried silt layer discourages the formation of Fe(III)-oxides within the HZ since it contains an enrichment of labile and mobile DOC which upwells into the intertidal mixing zone. This occurs under the influence of upwelling of deeper groundwater through the buried silt layer. The bacterial community within the sandy HZ may be further primed to degrade this DOC through the constant supply of riverine DOC within the regularly flushed intertidal zone sands. Thus, the electron donors required for the reductive dissolution of Fe(III)-oxides are maintained within the buried silt layer and just below ground surface (0.5 m) where riverbank muds accumulate at the end of each monsoon. In light of the totality of evidence presented from this and previous studies, we contend that if the buried silt layer was not present in the shallow aquifer, an NRB would form in the HZ as found at several other sites in permeable aquifers along tidally fluctuating rivers which did not have a buried silt layer (Figure 4a).^19,22,28,59^

Mobilization of DOC by upwelling of groundwater through the silt layer lowers the redox state in the HZ creating reducing conditions within the HZ such that an NRB cannot form at the end of the flow path. Instead, the upwelling reducing groundwater promotes reductive dissolution of pre-existing As-laden Fe(III)-oxides within silt and, to a lesser extent, in the overlying sand. The transport and dilution of these byproducts of reductive dissolution are evidenced by their increasing concentrations with depth as they approach the silt layer (Figure 3a,b,d,f). One could argue that the rapid increase in the concentrations of the byproducts of reductive dissolution of Fe(III)-oxides near the final 10 m of the flow path could be attributed to conservative transport of these byproducts within the upwelling groundwater (Figure 3a,b,d,f). Groundwater tends to upwell most intensely near the river edge according to theoretical groundwater flow models^42−46,61^ and this was confirmed by measured vertical hydraulic gradients on site. Then, this upwelling groundwater could convey the byproducts through the surficial riverbank sediment above the silt layer (Figure 4). Arsenic concentrations and byproducts of reductive dissolution increased with depth toward the bottom of the shallow Holocene aquifer as commonly seen across the delta.^3,5,62^ This alternative hypothesis of conservative transport of shallow aquifer water is rejected, however, on the basis of calculated excess element concentrations which utilized the results of the conservative mixing model (Text S7, Figure S14).^19,23^ This indicated that the byproducts of dissolution of Fe(III)-oxides were predominantly produced in situ within the buried silt layer (∼5 m deep) and the shallowest riverbank sediment (∼0.5 m deep) (Text S7, Figure S14). Other common processes that can drive As release include competitive desorption from the surfaces of Fe(III)-oxides by ions such as HCO_3_^–^, SO_4_^2–^, or phosphate (PO_4_^3–^).^63−67^ However, the observed concurrent release of dissolved Fe suggests that these processes are less likely the primary mechanism driving As release across the riverbank (Figure 3b).

The biogeochemical processes that regulate As mobility within sandy shallow riverbank aquifers with an intercalating silt layer has not previously been characterized (Figure 4). Our findings suggest that the presence of buried silt layers can significantly alter the dynamics of As mobility. The intercalating silt layer prevents As accumulation within the HZ on Fe(III)-oxides across intertidal zones, and instead generates a biogeochemical hotspot for As release within the HZ. In the presence of buried silt layers, riverbank aquifers within intertidal zones may therefore be susceptible to production of dissolved As in porewaters that adds to the mass flux of dissolved As advected from shallow Holocene aquifers toward the river.^22,53^ These findings modify the previous conceptual model in which permeable riverbank aquifers accumulate the As discharging to rivers. These findings expand our understanding of the fate of As discharging to rivers and the cycle of As across the broader shallow alluvial aquifers.

## Data Availability

The data sets generated or analyzed during this study are openly available in HydroShare at DOI: 10.4211/hs.a796954e87f04e7faff7b250660c6966. All other data sets generated and/or analyzed during the current study are also included in this published article (and its Supporting Information files).

## Supporting Information Available

Supporting Information including additional site information, detailed methods, and materials (PDF) is available. The Supporting Information file includes additional texts (Text S1–S8), figures (Figure S1–S14), and tables (Table S1–S7) (PDF)
Tidal fluctuations in a riverbank aquifer during dry season along the Meghna River, Bangladesh (AVI)

## Supplementary Material

es4c02330_si_001.pdf

es4c02330_si_002.avi

## Notes

All codes used as part of this study are publicly available. The statistical end-member mixing model SIMMR was used to statistically evaluate the mixing between three chemically distinct water bodies (https://andrewcparnell.github.io/simmr/).
