# Supplementary material for: Hotspots of Dissolved Arsenic Generated from Buried Silt Layers along Fluctuating Rivers
Source: Environ Sci Technol. 2024 Aug 13;58(34):15159–69. doi: 10.1021/acs.est.4c02330 (PMC11360370; doi:10.1021/acs.est.4c02330)
Supplement: Supplementary file 1 — es4c02330_si_001.pdf [file es4c02330_si_001.pdf]

Supplementary Information for

Hotspots of Dissolved Arsenic Generated from Buried Silt Layers along Fluctuating Rivers

Kyungwon Kwak[1], Thomas S. Varner[2], William Nguyen[3], Harshad V. Kulkarni[2,7], Reid Buskirk[1], Yibin Huang[1], Abu Saeed[4], Alamgir Hosain[5], Jacqueline Aitkenhead-Peterson[6], Kazi M. Ahmed[4], Syed Humayun Akhter[4], M. Bayani Cardenas[3], Saugata Datta[2], Peter S. K. Knappett[1]

[1] Department of Geology and Geophysics, Texas A&M University, College Station, TX 77843, USA

[2] Department of Earth and Planetary Sciences, The University of Texas at San Antonio, San Antonio, TX 78249, USA

[3] Department of Earth and Planetary Sciences, The University of Texas at Austin, Austin, TX 78712, USA

[4] Department of Geology, University of Dhaka, Dhaka 1000, Bangladesh

[5] Department of Coastal Studies and Disaster Management, University of Barishal, Barishal 8200, Bangladesh

[6] Department of Soil & Crop Science, Texas A&M University, College Station, TX 77845, USA

[7] School of Civil & Environmental Engineering, Indian Institute of Technology Mandi, Himachal Pradesh, 175005, India.

Corresponding author: Kyungwon Kwak; kkwak@tamu.edu

Summary:

Page S1-S43

Text S1-S8

Figure S1-S14

Table S1-S7

Video S1

Supplementary Information for

Hotspots of Dissolved Arsenic Generated from Buried Silt Layers along Fluctuating Rivers

Contents

Text S1. Site information and aqueous chemistry measurements
Text S2. Aquifer properties and hydraulic gradients
Text S3. Supplementary porewater chemistry profiles
Text S4. Stable water isotopes and ternary mixing between three water bodies
Text S5. Source of chloride (Cl^-) and bromide (Br^-)
Text S6. Electron transfer calculations, major chemical reactions, and arsenic speciation
Text S7. Excess concentration calculation to track the source of observed As concentrations
Text S8. The fate of arsenic advected to major rivers in deltas

Figure S1: Geologic cross-section and 2-D Electrical Resistivity tomogram
Figure S2. Three flow paths over which the number of electrons transferred were calculated
Figure S3: Hydraulic heads and their difference between river stage and MW3c
Figure S4: Hydraulic heads and their difference between MW1c and MW3c
Figure S5: Vertical hydraulic head differences in DP wells and time series of river stage
Figure S6: Supplementary porewater chemistry profile across the riverbank
Figure S7: Supplementary porewater chemistry profile for major cations, Si, and DIC
Figure S8: δD versus $\delta^{18}O$ plot of the water samples collected from all three well types
Figure S9: Monthly means of $\delta^{18}O$ composition of Dhaka precipitation
Figure S10: Three end-member mixing model from SIMMR model
Figure S11: Box plots of relative proportion of three end-members in wells from SIMMR model
Figure S12: Systematics of Cl/Br vs Cl^- Concentration to identify source of Cl^-
Figure S13: Arsenic speciation measurements in samples from river, DP, and MW wells.
Figure S14: Excess concentrations of redox-sensitive elements and dissolved carbon species

Table S1: Hydraulic conductivities (K) of MW and PZ wells calculated from slug tests
Table S2: Calculated relative proportions of three end-members in each well
Table S3: Relative proportions of three end-members in each well from SIMMR model
Table S4: Calculated number of electrons transferred across flow path from DP2b to DP6b
Table S5: Calculated number of electrons transferred across flow path from DP6b to river
Table S6: Calculated number of electrons transferred across flow path from DP1c to DP7c
Table S7: The key chemical reactions involving Fe and As cycling in the HZ of the riverbanks

Video S1: Tidal fluctuations in a riverbank aquifer along the Meghna River, Bangladesh

References

Text S1. Site information and aqueous chemistry measurements

Site selection and site information.

The location of the study site was chosen to establish an exemplary field site to assess the fate of dissolved As in shallow groundwater discharging to the river. A few key characteristics were sought. First, the shallow groundwater (<30 m) in this area had consistently high As concentrations. This was assessed by an exhaustive survey of dissolved As concentrations measured in 34,359 private wells in 2013 that spanned the entire north-south length of Araihazar along 15 km of the Meghna River (Figure 1a)[31]. Second, we sought a naturally sloping bank (i.e. not an abrupt artificial embankment) that leads to a well-defined riverbank, parts of which would be accessible over much of the year except during peak monsoon flooding (July-September) when the entire bank will be submerged. Banks that satisfy these first two criteria are common along the river but are punctuated by large stretches with low-lying fields that are inundated far inland from the river's dry season edge during the monsoon. Lastly, we sought a sandy bank to permit studying the chemical reactions that occur when the river water and groundwater advectively mix[19].

During the dry season, the exposed riverbank is approximately 131 m wide with a grade of 0.02 (2.6 m/131 m). Although the village itself rarely floods during the monsoon, the area around the village is located on the broad Meghna River floodplain which is inundated 2-3 km west (inland) during peak monsoonal flooding. This turns the villages into islands that are connected by narrow causeways. In the floodplain approximately 70% of ground elevations are less than three meters above sea level (masl) (Figure 1a). Although freshly re-worked sediment are annually deposited on the floodplain by the low-energy Meghna River, the sediment that comprise the 30 m deep shallow aquifer in this region were deposited by the higher energy Brahmaputra River during the Holocene period and are composed of sand and micaceous silt and clay[68].

In-situ aqueous chemistry measurements.

Each well (DP or MW) was pumped until temperature, pH, specific conductance (SC) and Oxidative-Reductive Potential (ORP) stabilized. The DP and PZ wells were pumped with a

peristaltic pump (Model 410, Solinst Canada Ltd., Georgetown, ON, Canada) with the manufacturer's low-flow adaptor and size 15 Masterflex tubing. The target flow rate was 50 mL/min, however, this flow rate varied as some DP wells recharged too slowly. These wells are colored red in Figure 1b. In these wells the pumping rate dropped as low as 10 mL/min. The MW wells were pumped using a plastic submersible pump (Typhoon Model, Groundwater Essentials LLC., Bradenten, FL) with a flow rate of approximately 2-4 L/min.

To observe the physical and chemical properties of the continuously flowing water, groundwater was pumped through a 350 mL flow-through cell (YSI Inc, Yellow Springs, OH) for the DP wells. For the MW wells, since the flow rate was higher a 5 L plastic bucket was utilized with the same multi-meter sensor (YSI Professional Plus, YSI Inc, Yellow Springs, OH) placed inside the bucket. The multi-meter was calibrated each morning. Redox-sensitive parameters which include dissolved oxygen (DO) (detection range: 0-1 ppm, Vacu-vials kit K-7553, CHEMetrics Inc., Midland, VA), nitrate (NO_3^-) (detection range: 0-50 ppm, Vacu-vials kit K-6933, CHEMetrics Inc., Midland, VA), ammonium (NH_4^+) (detection range: 0-60 ppm, Vacu-vials kit K-1413, CHEMetrics Inc., Midland, VA), sulfide (H_2S) (detection range: 0-3 ppm, Vacu-vials kit K-9503, CHEMetrics Inc., Midland, VA), manganese (Mn^{2+}) (detection range:0-2 ppm, CHEMets Visual kit K-6502, CHEMetrics Inc., Midland, VA), and iron (Fe^{2+}) (detection range: 0-10 ppm, CHEMets Visual kit K-6210, CHEMetrics Inc., Midland, VA) were sampled directly from the end of the tube with flowing water and measured on-site using colorimetric tests with a portable spectrophotometer (V-2000, CHEMetrics Inc., Midland, VA). Approximate concentrations of dissolved total As were measured in the field using a colorimetric arsenic test kit (Econo-Quick arsenic test kit, Industrial Test Systems Inc., Rock Hill, SC). Alkalinity was measured within 12 hours on samples that were kept on ice by titration with 1M H_2SO_4 and methyl red bromocresol green pH 5 indicator (Model AL-DT alkalinity test kit, HACH Company, Loveland, CO). The species HCO_3^- was assumed to comprise all the alkalinity in the near-neutral pH water, and from this were calculated moles of DIC.

Laboratory aqueous chemistry measurements.

All water samples to be analyzed by laboratory instruments were filtered on-site through a 0.45 μm nitrocellulose syringe filter (Millipore Millex – HP, Merck KGaA, Darmstadt,

Germany) using a plastic syringe into acid-cleaned 20 mL High Density Polyethylene (HDPE) vials that were pre-rinsed at least three times with the sample water. All syringes, HDPE vials, and tubing were pre-rinsed with 12 M of HCl prior to sampling. Major anions (Cl^-, Br^-, F^-, SO_4^{2-}, NO_3^-, NO_2^- and PO_4^{3}) were analyzed using ion chromatography (IC) (ICS-1000, Thermo Fisher Scientific, Waltham, MA). The anion samples were stored in a refrigerator at 4 ^{o}C and analyzed by IC at the Texas A&M University (TAMU) within 4 weeks of sampling.

Water samples for major cations (Ca^{2+}, K^+, Na^+, Mg^{2+}) and the redox-sensitive dissolved elements (Fe, Mn, and As) were acidified with Optima Grade HNO_3 (2% v/v). The samples were then analyzed using inductively coupled plasma spectroscopy (ICP-MS) (Element XR, Thermo Scientific, Waltham, MA) using an internal indium standard in the Williams Radiogenic Isotope Laboratory in the Department of Geology and Geophysics at TAMU. Water samples for As speciation measurements were filtered through an arsenic speciation cartridge packed with 2.5 g of aluminosilicate absorbent (Arsenic Speciation Cartridge, MetalSoft, Piscataway Township, NJ) to separate As(III) from total As (As(III) + As(V))[32,69,70].

Water samples for DOC analysis were filtered through 0.7 μm diameter ashed GF/F syringe filters (Whatman, Cytiva, Marlborough, MA) and stored in 40 mL amber glass vials that were pre-loaded with 20 μL of 12 M Optima grade HCl. The DOC samples were then stored in a refrigerator at 4 ^{o}C and analyzed as non-purgeable organic carbon (NPOC) by a total organic carbon analyzer (TOC-VCSH, Shimandzu, Kyoto, Japan) in the Department of Soil & Crop Science at TAMU within 2 weeks after sampling.

Stable water isotopes ($\delta^{18}O$ and δD) were analyzed by a cavity ring-down instrument, (Picarro L2120-i CRDS with AO211 vaporization module, Picarro Inc., Santa Clara, CA) in the Stable Isotope Geology Facility (SIGF) at TAMU. The samples were filtered on-site with a 0.45 μm nitrocellulose syringe filter (Millipore Millex – HP, Merck KGaA, Darmstadt, Germany) and refrigerated in tightly closed 20 mL clear glass vials. The isotopic measurements were calibrated to the internal standard "SIGF2013" ($\delta^{18}O$ = -4.71 ‰, δD = -27.4‰), which was calibrated using VSMOW (Vienna Standard Mean Ocean Water). The isotopic compositions were reported in standard δ–notation representing per mil (‰) deviations from the VSMOW standard.

Text S2. Aquifer properties and hydraulic gradients

Electrical resistivity imaging (ERI) and detailed borehole lithology observations from the three deepest monitoring wells (MWc wells) identified six major hydrogeologic units at the site[33]. From top to bottom these are: i) permanently saturated fine-sand layer above the intermediate silt layer, herein referred to as the riverbank aquifer; ii) transiently saturated fine-sandy layer; iii) leaky buried silt layer; iv) medium to course sand aquifer, herein referred to as the shallow Holocene aquifer; v) medium sand layer; and vi) a regionally widely present clay aquitard at 27 m depth which is commonly interpreted to divide the overlying Holocene and underlying pre-Holocene sediments (Figure S1)[33]. The DP wells and the MWa wells (~5 m depth) are screened within both the transiently and permanently saturated riverbank aquifer to characterize the evolution of groundwater chemistry along the flow path above and within the silt layer. The MWb (~10 m depth) and MWc wells (~20 m depth) are screened across the shallow Holocene aquifer to measure groundwater chemistry along the flow path below the silt layer.

Pressure transducers (Levelogger LT, Solinst Canada Ltd, Georgetown, ON, Canada) recorded the water levels in PZ and MW wells and river stage at 10 minutes frequency from January through October 2020. Submerged transducer pressure records were converted to water levels after correcting for changes in barometric pressure which was recorded by an on-site transducer (Barologger, Solinst Canada Ltd., Georgetown, ON, Canada). To measure the hydraulic conductivity (K) of the sediment falling head slug tests were performed on all PZ and MW wells using a pressure transducer that was lowered below the water level in the well. The transducer simultaneously acts as a mini slug while measuring the movement of the water level with high accuracy (<1 mm) and low frequency (0.25 seconds). The analytical solution by Bouwer and Rice[71] was used to solve for the K of the sediment.

The average K along the flow path above the buried silt layer, based on triplicate falling head slug tests performed in MWa wells using a pressure transducer and solid slug, was 20.4 m/day (range 4.3 to 30.1 m/day) whereas below the buried silt layer (MWb and MWc wells) was 44.1 m/day (range 26.9 to 53.1 m/day) (Table S1). The average K value of the monitoring wells (PZ wells) was 5.54 m/day (range 1.4 to 13.2 m/day) (Table S1).

The observed lateral hydraulic gradients amongst the MWc wells and the hydraulic head differences between MWc wells and river stage indicated that the Meghna River gains

groundwater for most of the year (Figure S3, Figure S4) at this location as found at three other sites nearby on both side of the river[22]. The river stage and the groundwater table are driven by semi-diurnal (12 hour) and neap-spring tides (14 day) during the dry season, whereas both are impacted by local and regional rainfall during the monsoon season (1 year)[19]. It is primarily these two processes of tides and local and regional rainfall that drive the interplay between the aquifer and the river which results in general seasonal patterns that favor groundwater flow towards the river for most of the year.

Closer to the river however, and during the dry season, sub-daily flow reversals do occur under the influence of strong tides that propagate 200 km upstream from the Bay of Bengal (Video S1 https://youtu.be/EqlDiIHC6Lw). The water table in MW3c was more sensitive to tidal river stage oscillations than the water table in MW1c owing to the attenuation of the pressure pulse (Figure S3, Figure S4)[17,72]. Tidal fluctuations were most pronounced during the dry season and the river even reverses the lateral flow direction within 50 m from the river's edge for several hours each day during high tide. This drives mixing between groundwater and river water within the aquifer across the intertidal zone[22]. According to the hydraulic head differences between DPb wells and river stage, the riverbank aquifer recharges the river most of the time during the dry season which agrees with the hydraulic head differences between MW wells and river stage (Figure S3, Figure S4, Figure S5, Video S1 https://youtu.be/EqlDiIHC6Lw).

The higher head conditions of the aquifer combined with tidal fluctuations also drive substantial vertical hydraulic gradients. To express this, simple head differences were calculated in the DP wells to determine the relative magnitude and direction of vertical groundwater flow along each flow path (Figure S5). Vertical K (K_y) was not measured in this study, so the magnitude of hydraulic gradients cannot be directly related to upward advective flow velocity (v). Hydraulic heads in the DP2, 3, 4, and 5 wells were measured using a hand-held water level meter approximately once every 30 minutes for 24 hours from January 14th to 15th, 2020. The hydraulic head differences between shallower DP well (either DPa or DPb wells) and deep DP well (DPc wells) reveal that the groundwater is upwelling at the end of the flow path through the buried silt layer during most of time of the day during dry season (Figure S5). The relative proportion of groundwater that travels upward vs. laterally cannot be calculated without knowing the distribution of K_y across the sands and silt layer but steepening vertical head gradients with

proximity to the river suggest that the rate of upwelling accelerates.

Text S3. Supplementary porewater chemistry profiles

While not a conservative tracer itself, the rising temperature along flow paths above the silt layer in the DPa and DPb wells with proximity to the river argues for upwelling of deeper 26 ºC groundwater through the silt layer at the dry season river edge since the river water is only 18 ºC and the average air temperature during the month of January in central Bangladesh ranges from a high of 25 ºC to a low of 14 ºC (Figure S6a). It is also noteworthy that the temperatures in the DPa and DPb wells increased laterally towards the river which further suggest an upwelling warm, deep groundwater from the flow path below the silt layer, although temperature is unlikely to behave conservatively across the flow path (Figure S6a). There is a caveat to this interpretation, however, the DPa and DPb wells closer to the river are screened at lower elevations as the riverbank slopes down. Therefore, wells at a given depth that are closer to the river are also closer to the underlying aquifer which has a year-round stable temperature of 26 ºC. The vertical differences in temperature, on the other hand, between the DPa, b, and c depths may indicate mixing with the cooler river water, or alternatively may be owing to the closer proximity of the screens to the cool ground surface. Based on the previously presented conservative mixing model and lateral and vertical hydraulic gradients, our interpretation of the observed temperature trends is that upwelling of warm groundwater is occurring, but mixing with the river is lowering the temperature of the porewaters in the 3-way river-riverbank aquifer-shallow aquifer mixing zone.

The specific conductance (SC) in the DP and MW wells ranged between 246 to 424 µS/cm, while the SC of the river was 120 µS/cm (Figure S6b). Even while mixing with the more dilute river, the SC increases in the DP wells with proximity to the river owing to the production of $HCO3^-$ which is the main anion contributing to SC.

The pH and oxidation-reduction potential (ORP) in the DP and MW wells ranged between 6.52 to 7.02 and -159 to 187 mV, respectively (Figure S6c,d). The pH and ORP of the river were 6.94 and 181 mV, respectively (Figure S6c,d). Negligible DO concentrations were observed in the DP and MW wells (<1 mg/L) (Figure S6e). Dissolved oxygen concentration of the river was over the detection limit (1 mg/L) (Figure S6e). Within the intertidal zone (DP wells), sulfate (SO_4^{2-}) concentration decreased towards the river (10 mg/L to 0.03) (Figure S6f).

Within the intertidal zone, dissolved silica (Si) concentration increased towards the river (8 to 17 mg/L), while the river contained relatively low Si concentration (4 mg/L) (Figure S7a). Concentrations of major cations (Ca^{2+}, K^{+}, Na^{+}, Mg^{2+}) increased towards the river (15 to 42 mg/L, 19 to 41 mg/L, 3 to 6 mg/L, and 6 to 14 mg/L, respectively) whereas the concentrations of major cations in the river were negligible (8, 1, 3, and 3 mg/L, respectively) (Figure S7b,c,d,e). The observed increasing trends in Si and major cations can be attributed to the oxidation of organic matter producing carbonic acid and subsequent silicate weathering in the riverbank aquifer[73]. By accepting a proton from carbonic acid, this process generates bicarbonate ions (HCO_3^-). The contribution of these bicarbonate ions to the DIC pool can be evidenced by the increasing trend of DIC observed across the intertidal zone (Figure S7f). The reductive dissolution of Fe(III)-oxides is the primary factor responsible for the production of DIC and consequently, the main cause of As and Fe release in the aquifer.

Text S4. Stable water isotopes and ternary mixing between three water bodies

Figure S8 displays the plot of δD versus $\delta^{18}O$ for the groundwater samples obtained from the DP, MW, and PZ wells. The groundwater samples were plotted along both the Local Meteoric Water Line (LMWL) and Global Meteoric Water Line (GMWL), characterized by the equations $\delta D = 7.7 * \delta^{18}O + 10.7‰$ and $\delta D = 8 * \delta^{18}O + 10‰$, respectively[74]. The groundwater samples collected from the three types of wells exhibit a distribution pattern that lies parallel and slightly below the GMWL and falls in between seawater and local river water isotopic compositions (Figure S8). This distribution indicates that the groundwater primarily originates from rainfall and/or flood water. The PZ wells, which are representative of groundwater samples across the water table, are influenced by evaporation as these points on Figure S8 plot farthest away from the GMWL. The MW wells plot close to the GMWL. This implies that the rainfall or flood waters that recharges the shallow aquifer experiences little evaporation as others have noted across the delta[37,74,75]. The DP wells occupy an intermediate position between the PZ and MW wells on the δD versus $\delta^{18}O$ plot. This can be attributed to the fact that the DP wells are screened at shallower depths compared to the MW wells and have a screen placement only 10 cm above the well bottom, which differs from the PZ wells' configuration. Figure S9 displays the time series of the average precipitation in Dhaka, along with the monthly means of $\delta^{18}O$ composition of the Dhaka precipitation. The isotopic composition of Dhaka precipitation is obtained from the Global Network of Isotopes in Precipitation (GNIP) by the International Atomic Energy Agency (IAEA). The average monthly $\delta^{18}O$ composition of Dhaka precipitation ranged from -8.47 to -0.39 ‰, with a noticeable depletion in isotopic composition during the monsoon season. The dry season river $\delta^{18}O$ composition is consistent with early monsoon season recharge to the shallow aquifer which supplies dry season baseflow to the river[22]. The shallow aquifer is recharged in the early dry season because the water table is still below ground surface[39], but by the middle of the monsoon the water table effectively rises to or above the ground surface (i.e., flooding).

A three end-member mixing model was developed to assess the proportionate contribution of three distinct water sources within the riverbank system. These sources include the river water, the riverbank groundwater, and the shallow Holocene groundwater (Figure S1, Figure 2d,e, Table S2). The model allows for the quantification of ternary mixing, providing

insights into the relative contributions of each water body within the riverbank system. The three end-members in the mixing model are i) riverbank groundwater (i.e., groundwater from the riverbank aquifer above the silt layer); ii) shallow Holocene groundwater (i.e., groundwater from the shallow Holocene aquifer below the silt layer); and iii) river water. The early monsoon river water was used to represent the shallow Holocene groundwater end-member. This is done because during the early monsoon river water recharges the riverbank water table. The river water end-member represents the surface water at the time of this study (dry season). In the mixing model, Cl^- concentration and value of $\delta^{18}O$ were assumed to be conservative tracers. The relative proportions of three end-members in groundwater were calculated using the mass balance equations below:

$$f_{sample} = 1 = f_1 + f_2 + f_3 \quad \text{Eq. S1}$$

$$\delta^{18}O_{sample} = f_1\delta^{18}O_1 + f_2\delta^{18}O_2 + f_3\delta^{18}O_3 \quad \text{Eq. S2}$$

$$Cl_{sample} = f_1 Cl_1 + f_2 Cl_2 + f_3 Cl_3 \quad \text{Eq. S3}$$

where the subscripts 1, 2, and 3 identify the three end-members (for this case, 1 = riverbank groundwater, 2 = shallow Holocene groundwater, and 3 = river water). By rearranging equations (S1)-(S3) to express them in terms of f_1 and f_2, the relative proportions of three end-members in each sample were calculated as below:

$$f_1 = \frac{Cl_{sample} - Cl_2 - f_3(Cl_3 - Cl_2)}{Cl_1 - Cl_2} \quad \text{Eq. S4}$$

$$f_2 = \frac{\delta^{18}O_{sample} - \delta^{18}O_1 - f_3(\delta^{18}O_3 - \delta^{18}O_1)}{\delta^{18}O_2 - \delta^{18}O_1} \quad \text{Eq. S5}$$

$$f_3 = 1 - f_1 - f_2 \quad \text{Eq. S6}$$

In addition, the Stable Isotope Mixing Model in R (SIMMR) was used to statistically evaluate the ternary mixing and quantify the relative proportions of three end-members in the groundwater (Figure S10, Figure S11, Table S3)[76]. SIMMR is a Bayesian mixing model that utilizes the mass conservation equation as described below[76–78].

$$X_{ij} = \sum_{k=1}^{p} g_{ik} f_{jk} + e_{ij} \quad \text{Eq. S7}$$

where *Xij* is the concentration of isotopic composition of species *j* of sample *i*. *p* is the number of assumed sources. f_{jk} is the tracer values of species *j* in source *k*. g_{jk} is the contribution of source *k* to sample *i*, and e_{ij} is the error yields. The SIMMR model employs a Markov chain Monte Carlo (MCMC) algorithm to optimize the fitting of the data. In this particular model, the following three end-members were selected: MW1a, MW2a, and MW3a represent the riverbank groundwater end-member. Additionally, two river water samples collected in January 2020 were chosen as the end-member for the river water end-member. Lastly, a river water sample collected in June 2015 was selected as the end-member for the shallow Holocene groundwater end-member. These selections serve as the basis for quantifying the relative proportions of the three end-members and assessing the ternary mixing in the riverbank system.

The relative proportions of three end-members in each well were calculated to evaluate the extent of ternary mixing occurring in the riverbank (Figure 2d,e, Table S2). Table S2 provides detailed information on the distribution and contribution of each end-member in the wells, offering insights into the overall mixing patterns within the riverbank.

Nest, the SIMMR was applied to statistically evaluate the ternary mixing and quantify the relative proportions of three end-members in the groundwater. In addition to conducting arithmetic calculations for determining the relative proportions of the three end-members in each well, the SIMMR model statistically described 90 percent of the DP and MW wells (Figure S10, Figure S11, Table S3). The model successfully distinguished the three end-members and provided a more rigorous and precise calculation of their relative proportions. These results further support the notion of robust mixing occurring among all three end-member water bodies, suggesting that the groundwater flow paths converge within and above the buried silt layer at the river's edge.

Text S5. Source of chloride (Cl^-) and bromide (Br)

Chloride concentrations below the silt layer were all below 10 mg/L and were similar to the river composition. At the water table above the silt layer, however, the concentrations exceeded 20 mg/L and two shallow DPs in the inter-tidal zone reached 50 mg/L. This suggests a source of dissolved Cl^- near the water table which means that Cl^- is not a purely conservative tracer. It appears to have a source, although it probably does not have a sink. This means there are some limitations on using it to quantify mixing between the riverbank aquifer groundwater and river water. The source of Cl^- in groundwaters was constrained using Cl^- concentrations and Cl^-/Br mass ratios (Figure S12). Owing to salts in human diets, leachates derived from sewage have higher Cl^-/Br mass ratio than natural organic leachates. In contrast, natural organic matter commonly has Cl^-/Br mass ratios lower than 200[54–56,79–82]. Thus, decomposition of natural organic matter concentrates Br relative to Cl^- in porewaters[56]. A mixing model of Cl^-/Br mass ratio was developed following the systematics utilized in a similar setting in a shallow aquifer in West Bengal by McArthur et al.[56] (Figure S12). Most of the groundwater samples (DP wells and MW wells) had Cl^-/Br mass ratio lower than 200, plotting below the seawater mixing line. This implies that the high Cl^- concentrations are primarily derived from decomposition of natural organic matter across the riverbank. This is supported by the observed increase in DOC as well as decomposition product of organic carbon like DIC and NH_4^+ across the intertidal zone.

Text S6. Electron transfer calculations, major chemical reactions, and arsenic speciation

The steep increase in DIC concentrations across the shallow flow path above the silt layer towards the river along with the increase in DOC concentrations is evidence of mobilization and/or oxidative mineralization of SOC (Figure 3e,f). The coincident rapid increase in dissolved reduced Fe(II) suggests that solid-phase Fe(III) is an important EA. The number of electrons transferred during the oxidation/mineralization of DOC was calculated to determine the major redox reactions driving the local distributions of dissolved As and Fe in the riverbank aquifer (Figure S2, Table S4, Table S5, Table S6)[41]. Detailed descriptions of methods for the electron transfer calculations can be found in the method section.

Table S4 presents the electron transfer calculations along the flow path segment above the silt layer from the DP2b to DP6b. This segment extends from the well with lowest concentrations of dissolved Fe and As to the well with highest concentrations. The same calculations were performed for other flow path segments, including DP6b to river, which extends from the well most proximal to the river to the river above the silt layer (Table S5). Lastly, the pathway within the silt layer from DP1c to DP7c which is from the well furthest from the river to the well nearest to the river was constrained (Table S6). Notably, the available ED (0.03 meq/L) and EA (-1.07 to -0.01 meq/L) in the aqueous phase at the start of the flow path were inadequate to account for the total number of electrons that were transferred during DOC mineralization (4.66 meq/L) across the DP2b to DP6b flow path as evidence by DIC production (Table S4). The results were similar across the two other flow paths (DP6b to river and DP1c to DP7c; Table S5 and S6, respectively). Given that the mineralization of one mole of DOC produces one mole of DIC, these results indicate that SOC (ED) and Fe(III)-oxides (EA) along the flow paths in the riverbank must be utilized by bacteria to produce the observed high DIC concentrations.

Table S7 summarizes the major chemical reactions that are proposed to drive Fe and As cycling in the HZ of the riverbanks under dynamic flow conditions: i) SOC dissolution to DOC, ii) aerobic respiration of DOC, iii) abiotic oxidation of dissolved Fe(II), iv) microbially-mediated reductive dissolution of Fe(III)-oxides which is coupled with DOC mineralization, and v) adsorption/desorption of As to/from the surfaces of Fe(III)-oxides.

Figure S13 presents the As speciation measurements in water sample from river, DP, MW wells to separate As(III) from total As (As(III) + As(V)). Approximately 90 percent of the water samples plotted on or slightly below the 1:1 line. This indicates that As(III) is the dominant As species, consistent with findings from previous studies conducted in Bangladesh aquifers[28,32,70].

Text S7. Excess concentration calculation to track the source of observed As concentrations

Based on the hydrology and the aqueous chemistry data presented in this study along with the sedimentary chemistry data presented in Varner et al.[14,57], the potential sources contributing to the observed concentration of dissolved As can be summarized as follows: i) riverbank sediment; ii) buried silt layer; and iii) upwelling groundwater from the high-As shallow Holocene aquifer. To identify the most significant depths where the products are produced within the intertidal zone, excess concentrations of redox-sensitive ions and elements (As, Fe, NH4+, DOC, and DIC) were calculated.

Once the relative proportions of three end-members are calculated along the flow path (Text S4), the excess concentrations of a relevant ion or element are calculated to account for dilution of the riverbank groundwater within and above the buried silt layer, which is a mixture of groundwater from underlying Holocene aquifer and the shallow riverbank aquifer, by river water. This calculation estimates the cumulative concentration of each element produced or consumed relative to values expected if all of the observed chemistry was produced by conservative transport and mixing.

After calculating the relative fractions of each end-members from equations (S1)-(S6) (Text S4), the excess concentration of a relevant ion or element can be calculated using the following equation[19,23]:

$$[X]_{excess} = [X]_{sample} - ([X]_1 f_1 + [X]_2 f_2 + [X]_3 f_3) \quad \text{Eq. S8}$$

where $[X]_{excess}$ is the excess concentration of the relevant element within a given well along the flow path. $[X]_{sample}$ is the observed concentration of the relevant element in the same well. The terms $[X]_1$, $[X]_2$, $[X]_3$ represent the concentration of the relevant element of each end-member. The terms f_1, f_2, f_3 represent the relative proportion of each end-member of the sample. The subscripts 1, 2, and 3 represent the end-members. In the excess concentration calculation, the MW3a, MW3c, and river water samples were chosen as the end-members to account for dilution in the shallow intertidal zone.

Figure S14 presents the calculated excess concentrations of redox sensitive elements across the shallow intertidal zone. Excess As concentrations gradually increased from -44 to 30 µg/L across the flow path (Figure S14a). Dissolved As was consumed between 30 to 10 m from

the river, but then the excess As increased ~50 µg/L between 10 to 0 m from the river up to 30 µg/L (Figure S14a). The excess As concentrations were highest in the DPa (~0.5 m deep) and DPc (~5 m deep) wells along the final 10 m of the flow path (8 to 17 µg/L and 12 to 30 µg/L, respectively). However, dissolved As was either consumed or conservatively transported in the DPb (~1.5 m deep) wells across the entire shallow intertidal zone. This indicates that the dissolved As was predominantly produced within the shallowest riverbank sediment (~ 0.5 m deep) and the buried silt layer (~5 m deep), rather than within the intermediate riverbank sediment (~1.5 m). Similarly, substantial quantities of dissolved Fe were produced within the shallowest riverbank sediment (1118 to 2347 µg/L) and the buried silt layer (1535 to 2523 µg/L) along the flow path (Figure S14b). Except for DP6b well, the dissolved Fe was transported conservatively across the flow path of 1.5 m depth. Dissolved NH_4^+ was predominantly produced within the buried silt layer, while the excess NH_4^+ increased towards the river regardless of the depths of the flow path (Figure S14c). DOC was consumed across the entire flow path, however, the excess DOC increased from ~0 to 2 mg/L along the final 10 m of the flow path (Figure S14d). Similar to dissolved As, Fe and NH_4^+, substantial quantities of DIC were produced within the very shallow riverbank sediment and the buried silt layer along the entire flow path up to 136 mg/L (Figure S14e).

These results collectively indicate that the observed concentrations of As and Fe, and other by-products of dissolution of Fe(III)-oxides such as NH_4^+, DIC were primarily produced in-situ within the surficial riverbank sediment just below the surface (~0.5 m deep) and within the buried silt layer (~5 m deep). This is in addition to As carried by upwelling groundwater, which was a minor component. The As-laden Fe(III)-oxides present within the shallow riverbank sediment just below the surface and buried silt layer experience microbially-mediated reductive dissolution using the locally-produced labile DOC as ED, acting as a biogeochemical hotspot for dissolved As and Fe across the shallow intertidal zone. These findings are consistent with findings from Varner et al.[14,57] where the authors reported that the buried silt layer and riverbank sediment contain high proportions of labile organic matter that promote reductive dissolution of Fe(III)-oxides, preventing As accumulation along the shallow intertidal zone.

Text S8. The fate of arsenic advected to major rivers in deltas

The fate of dissolved As within the Holocene aquifer that is advected from upgradient is unclear. Although the shallow groundwater discharges to the Meghna River for most of the year, the river water contains very low concentrations of dissolved As (4 µg/L) and Fe (2 µg/L) even under baseflow conditions during the dry season where typical regional shallow aquifer As concentrations are over 200 µg/L[22]. When the river is losing, our data collected in the dry season suggests that the shallow intertidal HZ remains under an Fe-reducing redox state with few sorption sites (available surfaces of Fe(III)-oxides) for As to re-adsorb to. Possible scenarios are that the As concentrations are simply diluted to such low levels with large rivers, however, during prolonged dry season most of the river would be comprised of baseflow that has high As concentrations[83].

Recent studies have suggested that in a sufficiently permeable riverbank a NRB forms 1-5 m below the river-aquifer interfaces[19,28,32]. We suspect that, at this site, there is an As sink across the final decimeter of the permanently below the water table transport path to the river when the reduced Fe, As mix with oxygen from the river rapidly precipitating Fe(III)-oxides. The picture in Figure 1c with the orange layers suggests this is the NRB at this site.

The locus of accumulation of solid-phase As is crucial. If the Fe(III)-oxides and As form within 5-10 cm of the riverbed, this material could easily be scoured during the monsoon and redeposited on floodplains. In this way, solid-phase As may be transported down the delta in the river's suspended load, be deposited as overbank deposits, reductively dissolved, and travel back towards the river to either be trapped in a high K buried NRB, or a more surficial NRB that may be easily scoured. Larsen et al.[29] reported that a considerable amount of dissolved As was co-deposited with reactive Fe(III)-oxides and integrated into the river sediment on the floodplain of the Red River in Vietnam. They discussed that these As and Fe-rich sediment are likely to be transported downstream and re-deposited along the banks of the river, forming a new layer of aquifer over time. Here we present evidence of this process operating along the Meghna River. The As and Fe-rich riverbank sands either represent iron oxides that developed in-situ on site from dissolved Fe and As or the overbank deposits upstream may be re-deposited along the intertidal zones. The re-cycling of As and Fe then continues along the river shorelines down the delta until ultimately reaching the ocean.

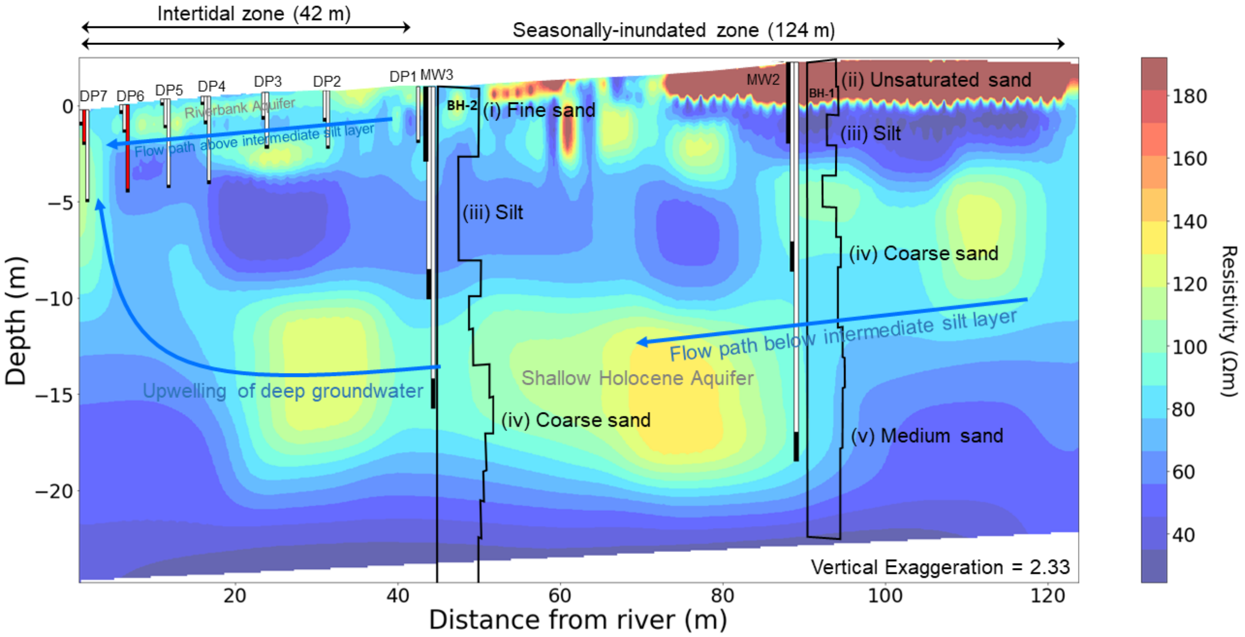

Figure S1. Geologic cross-section and 2-D Electrical Resistivity tomogram and layout of DP and MW wells. Screened intervals are shown in black. There are six hydrogeologic units. From top to bottom these are: i) ~2 m thick fine-sandy vadose zone that is represented as a high resistivity zone (>180 Ωm); ii) ~3 m thick saturated fine to medium sandy layer, herein referred to as the riverbank aquifer (100 to 140 Ωm); iii) ~4 to 5 m thick leaky silt aquitard, herein referred to as the buried silt layer (~40 to 60 Ωm); iv) ~12 m of medium to coarse sand aquifer, herein referred to as the shallow Holocene aquifer (140 Ωm); v) ~7 m of medium sand (~100 Ωm); and vi) a regional blue clay (high plasticity) aquitard at ~27 m depth. The DP wells and the MWa wells (~5 m depth) are screened across the riverbank aquifer above the buried silt layer. The MWb and MWc wells (~10 m depth and ~20 m depth, respectively) are screened across the shallow Holocene aquifer below the buried silt layer. The DP wells that were not functioning were filled with red. The MW1 wells are located at the 131 m position. Modified from Pedrazas et al.[33].

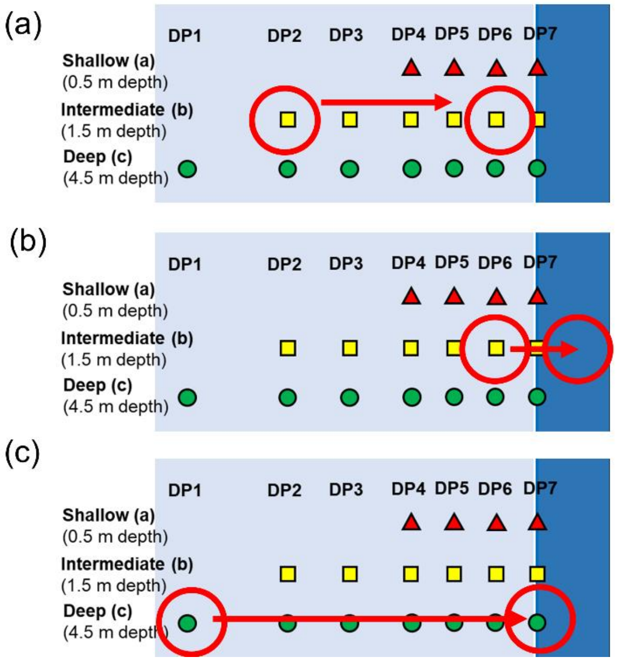

Figure S2. Three flow paths over which the number of electrons transferred were calculated. (a) DP2b to DP6b (from the well with lowest concentrations of Fe and As to the well with highest concentrations); (b) DP6b to the river; (c) DP1c to DP7c (from the well furthest from the river to the well nearest to the river).

483

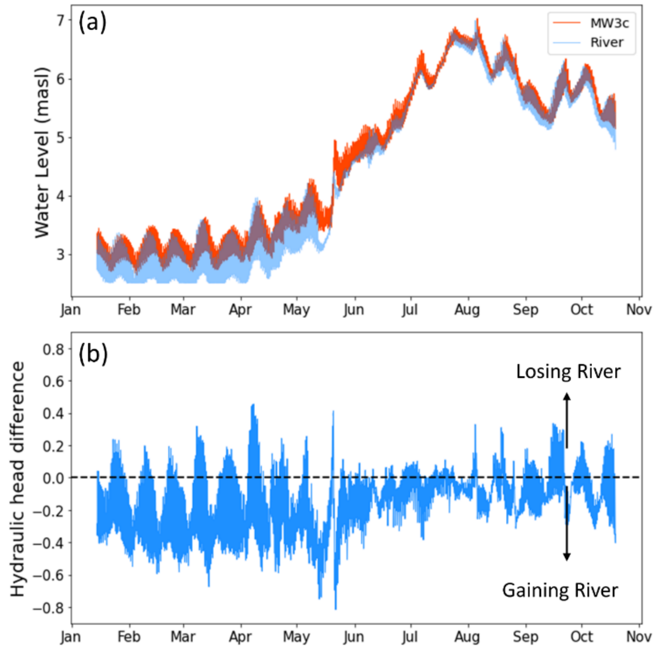

Figure S3. Hydraulic heads and their difference between river stage and monitoring well MW3c from January 2020 to October 2020. (a) Hydraulic heads of MW3c and Meghna River. (b) Difference in hydraulic heads between MW3c and the river. Positive and negative hydraulic head differences correspond to losing and gaining conditions of the Meghna River, respectively.

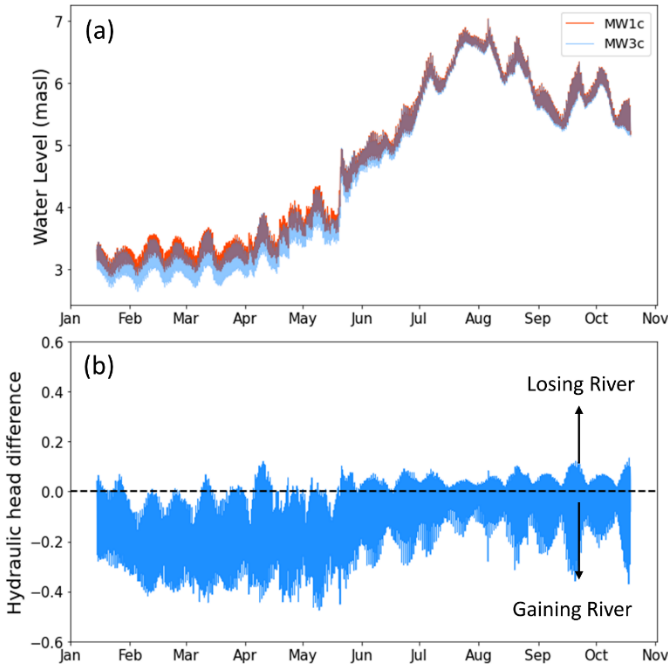

Figure S4. Hydraulic heads and their difference between MW1c and MW3c from January 2020 to October 2020. (a) Hydraulic heads of MW1c and MW3c. (b) Difference in hydraulic heads between MW1c and MW3c. Positive and negative hydraulic head differences correspond to losing and gaining conditions of the Meghna River, respectively.

487

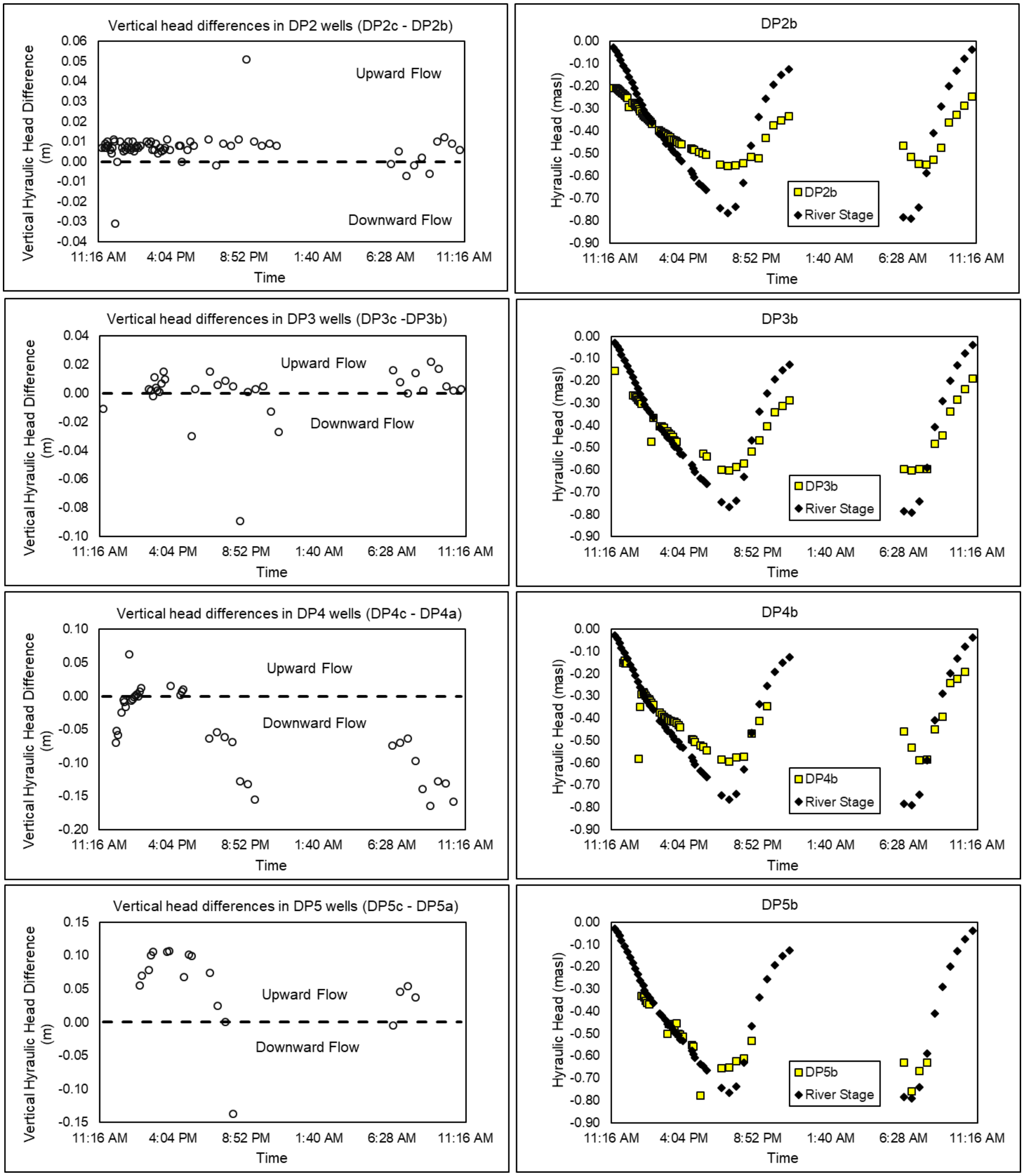

Figure S5. Vertical hydraulic head differences in DP wells and time series of river stage. Hydraulic heads in the DP well nests were measured manually approximately every 30 minutes, except at nighttime, with hand-held water level meter over 24 hours from Jan 14 to Jan 15, 2020. Positive vertical head difference indicate that the groundwater flows upward, and negative difference indicates that the groundwater flows downward at the end of the flow path. The head differences in DP2, DP3, and DP5 wells indicate that the groundwater is upwelling across the intertidal zone during most of time of the day in dry season. River stages were measured using pressure transducers.

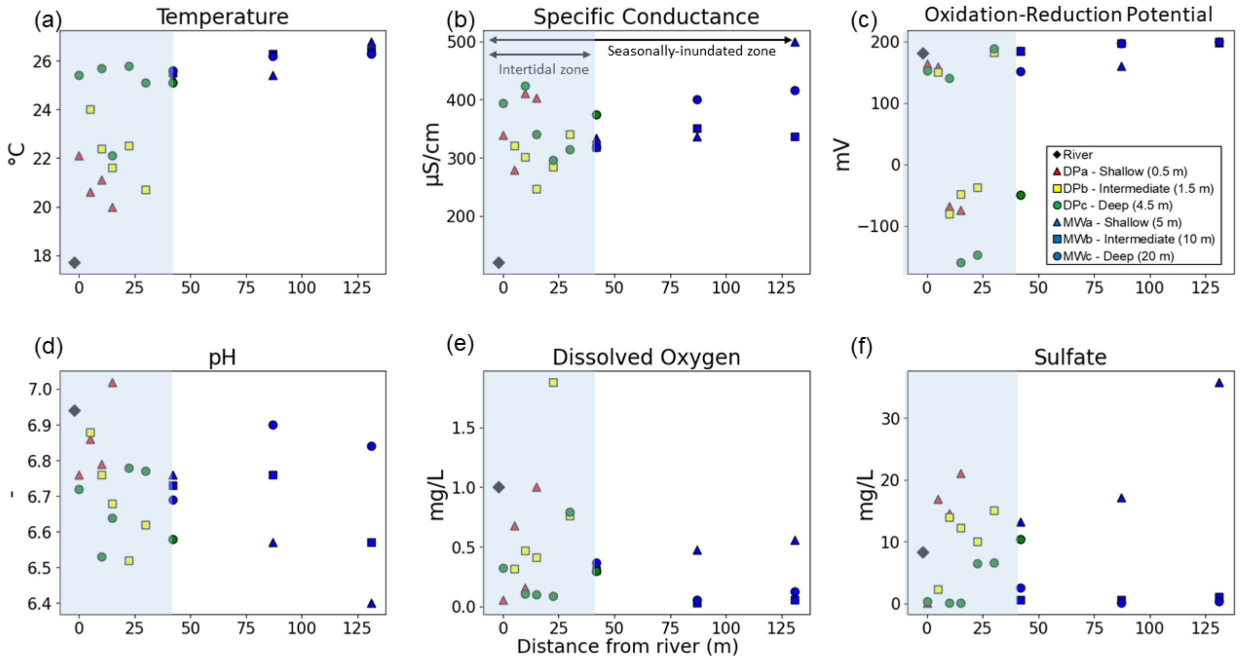

Figure S6. Supplementary porewater chemistry profile across the intertidal zone (blue box) and seasonally-inundated zone across the riverbank. (a) temperature, (b) specific conductance (SC), (c) oxidation-reduction potential (ORP), (d) pH, (e) dissolved oxygen (DO), and (f) sulfate (SO_4^{2-}) concentrations are plotted. Red triangles, yellow squares, and green circles represent shallow (~0.5 m), intermediate (~1.5 m), and deep (~3 to 4.5 m) DP wells respectively. Blue triangles, squares, and circles represent shallow (~5 m), intermediate (~10 m), and deep (~20 m) MW wells. The black diamond represents river water composition. The x axis describes the lateral distance of each well from the dry season river shoreline during low, neap tide.

490

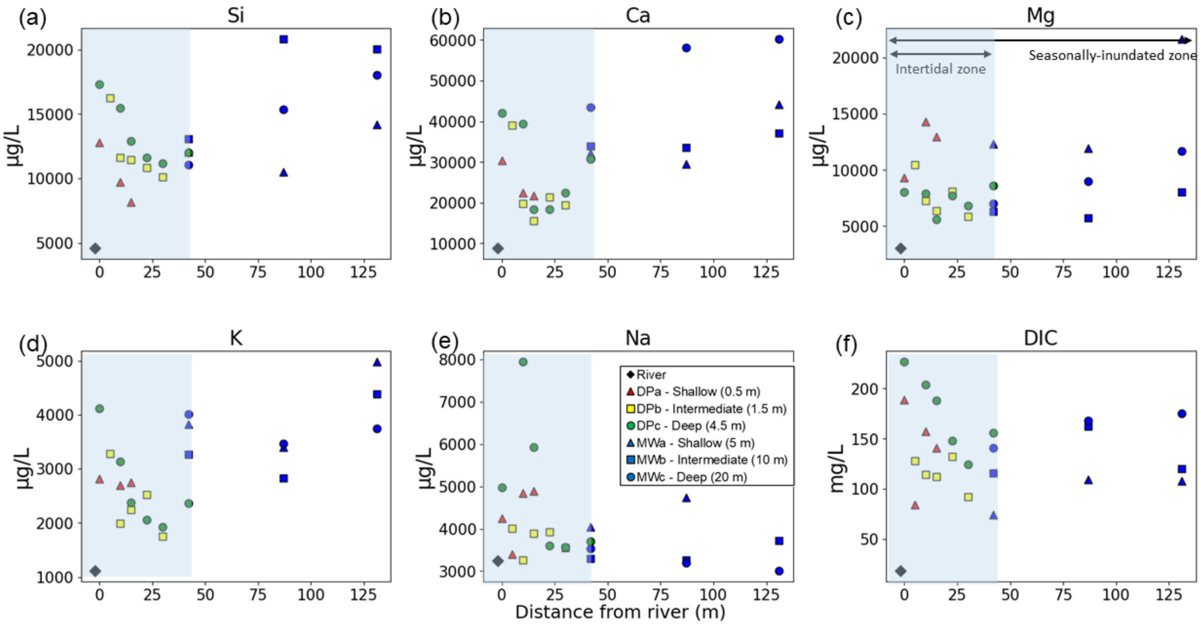

Figure S7. Supplementary porewater chemistry profile for major cations (Ca, Mg, K, Na), Si, and DIC across the intertidal zone (blue box) and seasonally-inundated zone across the riverbank. (a) dissolved silica (Si), (b) calcium (Ca), (c) magnesium (Mg), (d) potassium (K), (e) sodium (Na), (f) DIC are plotted. Red triangles, yellow squares, and green circles represent shallow (~0.5 m), intermediate (~1.5 m), and deep (~3 to 4.5 m) DP wells respectively. Blue triangles, squares, and circles represent shallow (~5 m), intermediate (~10 m), and deep (~20 m) MW wells. The black diamond represents river water composition. The x axis describes the lateral distance of each well from the dry season river shoreline during low, neap tide.

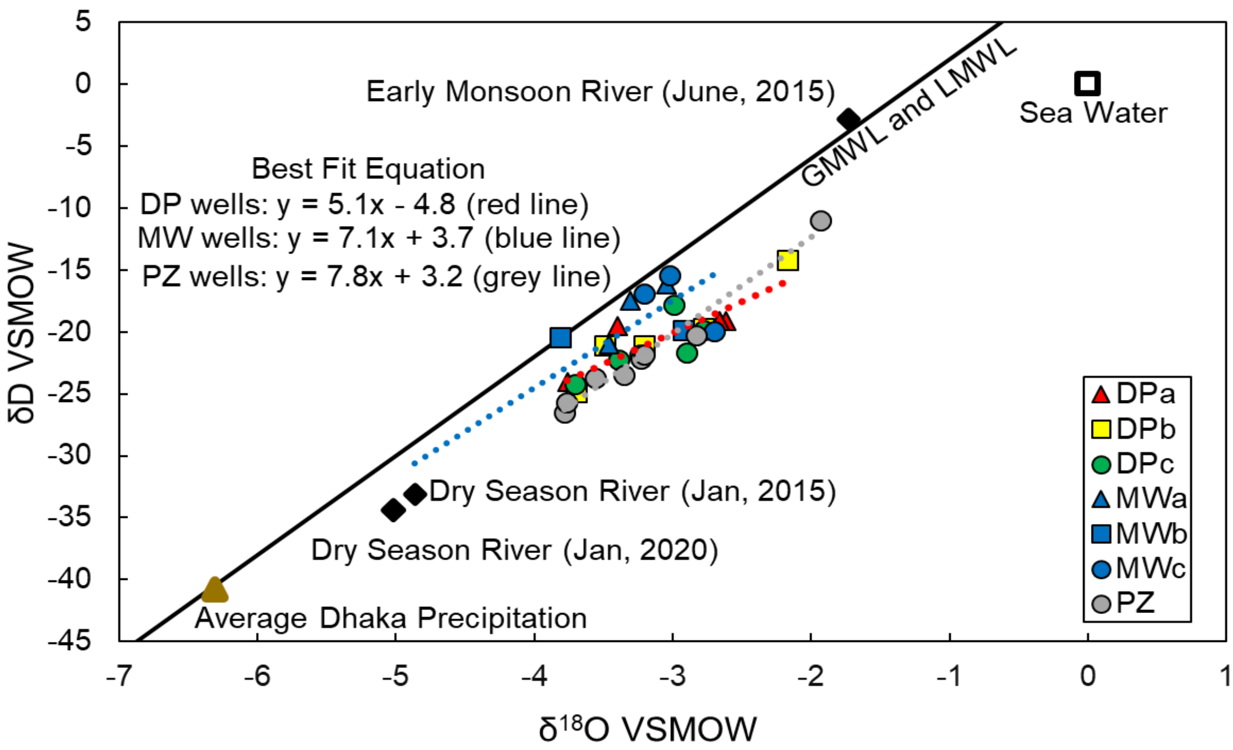

Figure S8. δD versus $\delta^{18}O$ plot of the water samples collected from all three well types (DP, MW, and PZ). The Local Meteoric Water Line (LMWL) and Global Meteoric Water Line (GMWL) have equations of $\delta D = 7.7 * \delta^{18}O + 10.7‰$ and $\delta D = 8 * \delta^{18}O + 10‰$, respectively. The LMWL and GMWL are from Majumder et al.[74]. The average isotopic composition of Dhaka precipitation is from the Global Network of Isotopes in Precipitation (GNIP) by the International Atomic Energy Agency (IAEA).

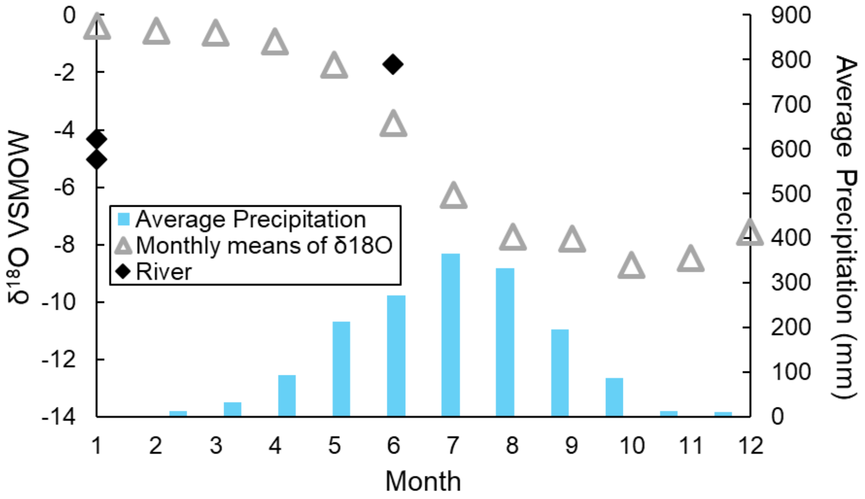

Figure S9. Monthly means of $\delta^{18}O$ composition of Dhaka precipitation. Average precipitation and the $\delta^{18}O$ composition of Dhaka precipitation are from GNIP by the IAEA. Three observations of the Meghna River water are presented. Two dry season river water samples are collected in January, 2015 and January, 2020, respectively. One early monsoon river water sample was collected in June, 2015.

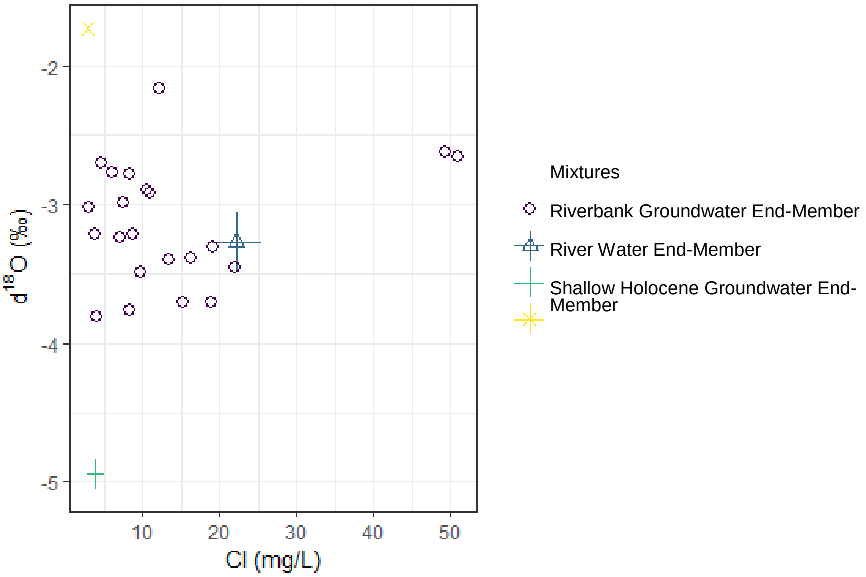

Figure S10. Three end-member mixing model from SIMMR. MW1a, MW2a, and MW3a represent the riverbank groundwater end-member. Two river water samples collected in January 2020 were chosen as the end-member for the river water. One river water sample collected in June 2015 was selected as the end-member for the shallow Holocene groundwater. The mixing model described approximately 90 percent of the DP and MW wells.

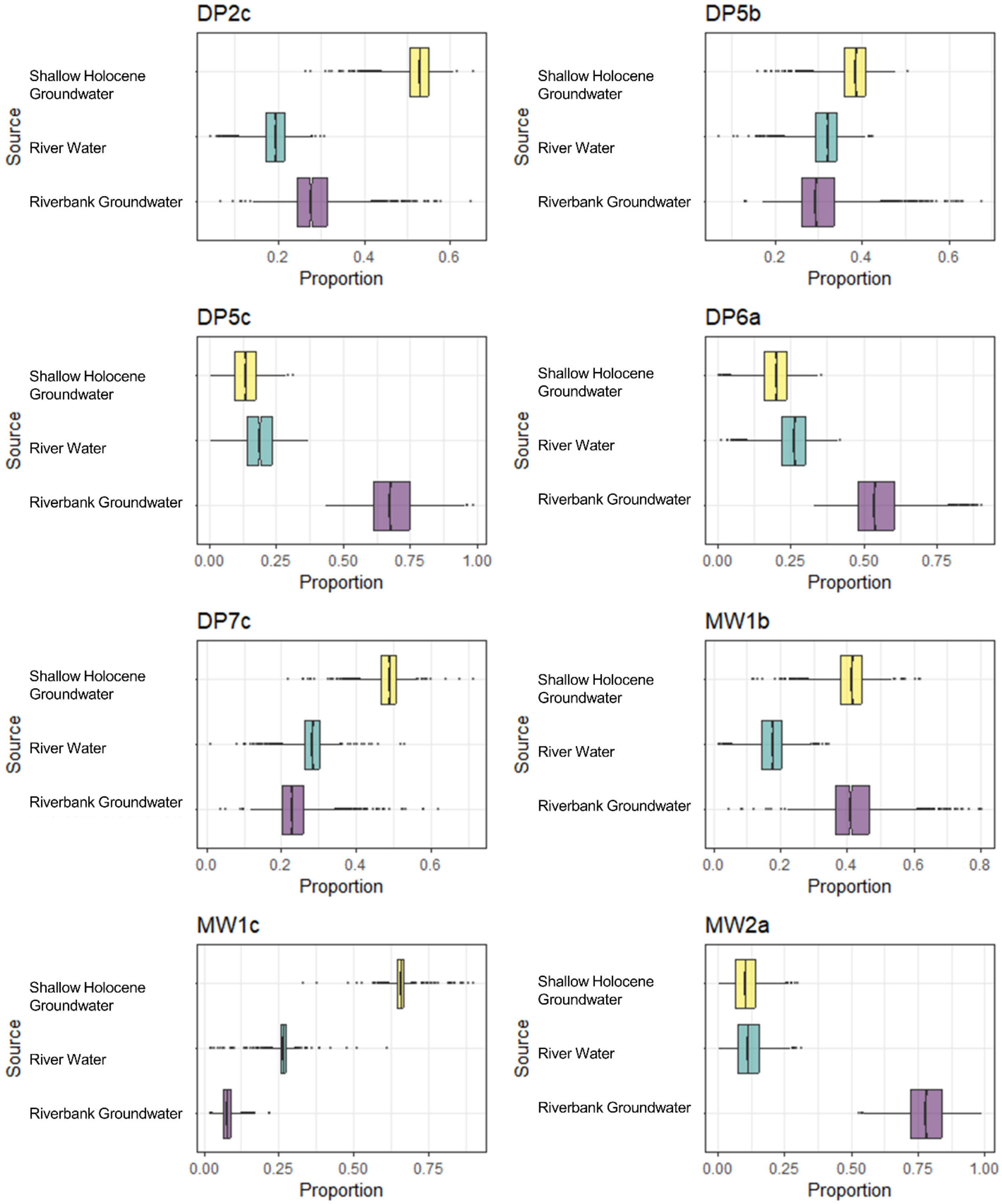

Figure S11. Box plots of relative proportion of three end-members in each DP and MW wells from SIMMR model.

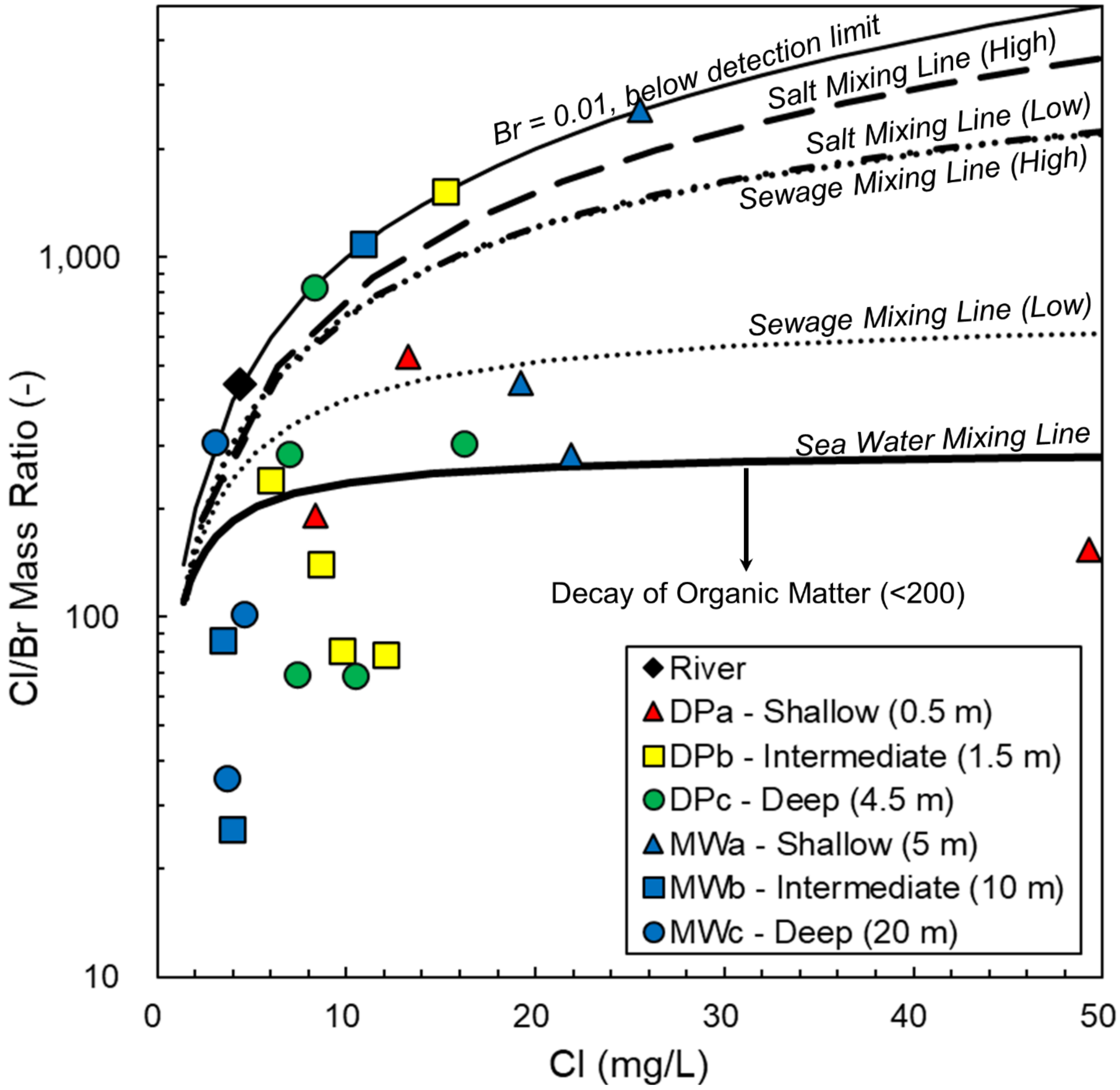

Figure S12. Systematics of Cl/Br vs Cl^- Concentration to identify source of Cl^- following McArthur et al.[56].The sewage mixing lines are from Vengosh and Pankratov[82] and McArthur et al.[56]. The halite and sea water mixing line were calculated following Alcala and Custodio[55]. Rainwater in the West Bengal has Cl^- concentration of 1.4 mg/L and Cl^-/Br mass ratio of 110[56]. The thin black solid line above the high salt mixing line represents the samples with Br concentrations that are lower than the detection limit of the IC (0.01 mg/L). Therefore, the true position of these points lies above the high-end member salt mixing line.

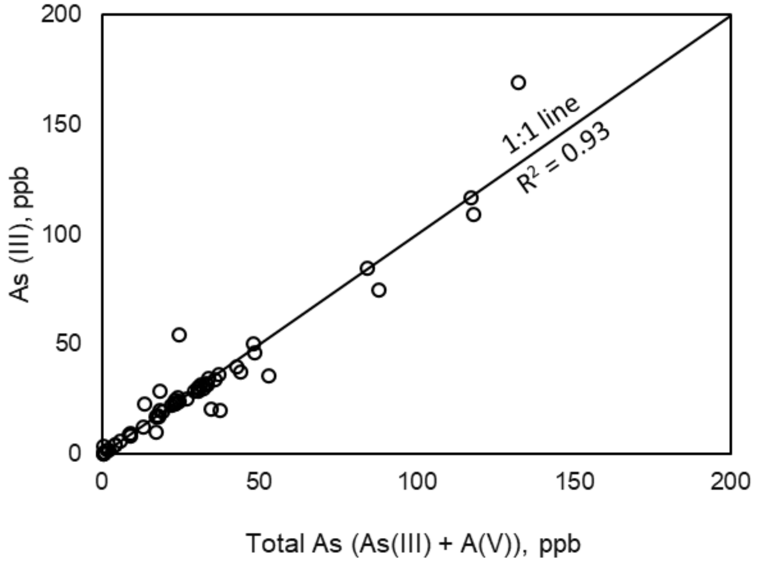

Figure S13. Arsenic speciation measurements in water samples from river, DP, and MW wells. Total As (As(III)+As(V)) concentrations were plotted against As(III) concentrations.

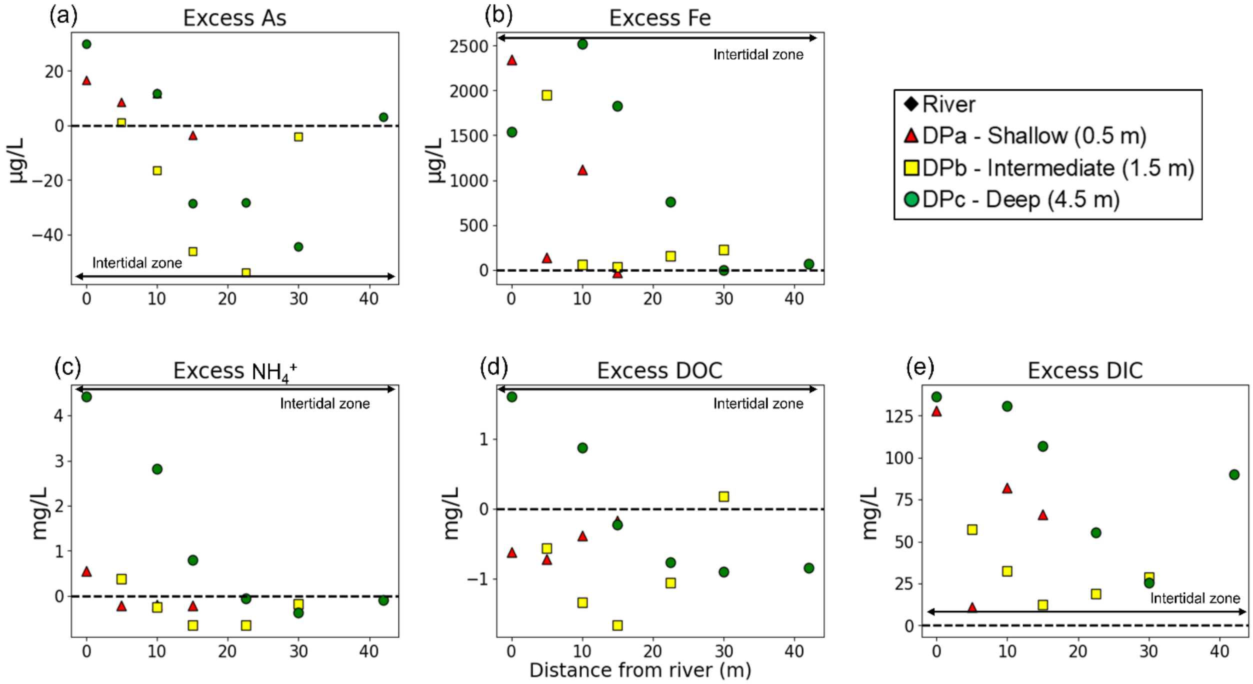

Figure S14. Calculated excess concentrations of redox-sensitive elements (a-c) and dissolved carbon species (d-e) in the shallow intertidal zone. Red triangles, yellow squares, and green circles represent DPa (~0.5 m), DPb (~1.5 m), and DPc (~3 to 4.5 m) wells, respectively. The x axis describes the distance of each well from the dry season river shoreline during low, neap tide. The MW3a, MW3c, and river water samples were used as three end-members to account for dilution of riverbank groundwater with upwelling shallow Holocene groundwater and river water.

Table S1. Hydraulic conductivities (K) of MW and PZ wells calculated from slug tests

Well	Slug test #	K (m/day)	Average K (m/day)	Standard Deviation	Coefficient of Variation (%)
MW1a	1	27.15	26.7	6.3	23
	2	32.75			
	3	20.24			
MW1b	1	47.69	48.8	1.5	3
	2	49.87			
MW1c	1	59.15	49.5	13.7	28
	2	39.74			
MW2a	1	4.56	4.3	0.3	8
	2	4.08			
MW2b	1	40.59	34.9	8.0	23
	2	29.24			
MW2c	1	54.90	51.6	4.7	9
	2	48.29			
MW3a	1	28.58	30.1	7.0	23
	2	37.77			
	3	24.07			
MW3b	1	35.33	53.1	21.0	40
	2	47.69			
	3	76.30			
MW3c	1	24.54	26.9	3.4	13
	2	29.35			
PZ1	1	0.50	1.4	1.0	72
	2	1.20			
	3	2.50			
PZ2	1	5.80	5.8	0.0	0
	2	5.80			
PZ3	1	5.60	5.3	0.3	7
	2	5.10			
PZ4	1	13.20	13.2		
PZ6	1	2.00	2.0		

Table S2. Calculated relative proportions of three end-members in each well. Fraction of the three end-members was calculated using $\delta^{18}O$ and Cl^- concentration. MW1a represents the riverbank groundwater end-member. Dry season river represents the river water end-member. Early monsoon river represents the shallow Holocene groundwater end-member.

Well	$\delta^{18}O$ (‰ $_{VSMOW}$)	Cl (mg/L)	Fraction (-)		
			Shallow Holocene Groundwater	Riverbank Groundwater	River Water
DP1c	-3.70	18.841	-0.01	0.68	0.33
DP2b	-3.70	15.206	0.09	0.52	0.39
DP2c	-2.78	8.288	0.55	0.22	0.23
DP3b	-2.17	12.061	0.62	0.41	-0.03
DP3c	-2.90	10.468	0.45	0.32	0.23
DP4a	-2.62	49.288	-0.52	2.08	-0.56
DP4b	-2.77	5.955	0.61	0.12	0.27
DP4c	-3.24	6.976	0.45	0.15	0.40
DP5a	-2.66	51.006	-0.59	2.16	-0.58
DP5b	-3.21	8.667	0.41	0.23	0.36
DP5c	-3.39	16.188	0.15	0.57	0.28
DP6a	-3.40	13.301	0.23	0.44	0.33
DP6b	-3.49	9.799	0.30	0.28	0.42
DP7a	-3.76	8.378	0.26	0.21	0.54
DP7c	-2.99	7.406	0.51	0.18	0.31
PZ1	-1.93	1.898	0.97	-0.05	0.08
PZ2	-2.83	19.213	0.23	0.72	0.05
PZ3	-3.78	15.664	0.05	0.54	0.41
PZ4	-3.35	24.345	-0.06	0.94	0.12
PZ5	-3.23	31.837	-0.23	1.28	-0.06
PZ6	-3.77	20.334	-0.07	0.75	0.32
PZ7	-3.56	42.094	-0.60	1.74	-0.14
PZ8	-3.21	36.730	-0.35	1.50	-0.15
VHGW	-3.75	16.113	0.05	0.56	0.39
MW3a	-3.45	21.884	-0.02	0.83	0.20
MW3b	-3.81	3.981	0.36	0.01	0.63
MW3c	-3.21	3.703	0.55	0.01	0.45
MW2a	-3.31	19.191	0.10	0.71	0.20
MW2c	-3.02	3.067	0.62	-0.02	0.40
MW1a	-3.04	25.547	0.00	1.00	0.00
MW1b	-2.92	10.861	0.43	0.34	0.23
MW1c	-2.70	4.548	0.67	0.06	0.27
Dry Season River	-5.02	4.404	0.00	0.00	1.00
Early Monsoon River	-1.73	2.89	1.00	0.00	0.00

Table S3. Calculated relative proportions of three end-members in each well from SIMMR model. Fraction of the three end-members was calculated using $\delta^{18}O$ and Cl^- concentration.

Well ID	Riverbank Groundwater		River Water		Shallow Holocene Groundwater	
	mean	standard deviation	mean	standard deviation	mean	standard deviation
DP1c	0.719	0.082	0.22	0.074	0.061	0.032
DP2b	0.601	0.082	0.307	0.067	0.092	0.042
DP2c	0.284	0.053	0.191	0.032	0.525	0.034
DP3b	0.378	0.043	0.02	0.01	0.601	0.044
DP3c	0.402	0.073	0.172	0.043	0.425	0.048
DP4a	0.977	0.011	0.01	0.007	0.013	0.008
DP4b	0.156	0.033	0.25	0.021	0.594	0.022
DP4c	0.206	0.046	0.372	0.03	0.421	0.03
DP5a	0.979	0.01	0.009	0.006	0.012	0.008
DP5b	0.3	0.062	0.317	0.038	0.382	0.039
DP5c	0.68	0.094	0.184	0.065	0.136	0.057
DP6a	0.553	0.097	0.254	0.062	0.194	0.059
DP6b	0.358	0.071	0.377	0.045	0.264	0.045
DP7a	0.277	0.059	0.5	0.039	0.223	0.039
DP7c	0.235	0.049	0.28	0.031	0.484	0.031
MW3a	0.829	0.067	0.105	0.054	0.065	0.038
MW3b	0.049	0.024	0.626	0.025	0.325	0.025
MW3c	0.038	0.018	0.443	0.018	0.519	0.018
MW2a	0.777	0.082	0.117	0.056	0.106	0.053
MW2c	0.021	0.011	0.392	0.014	0.586	0.015
MW1b	0.42	0.074	0.171	0.043	0.409	0.05
MW1c	0.08	0.02	0.264	0.014	0.656	0.015

Table S4. Calculated number of electrons transferred during organic carbon oxidation across flow path from DP2b to DP6b.

Electron Donors (ED)	Electrons Transferred Per Reaction (mol)	DP2b (mmol)	DP6b (mmol)	DP2b - DP6b (mmol)	Electrons Transferred (meq/L)
DOC	-4	0.04	0.03	0.01	0.03
Electron Acceptors (EA)					
Fe (III)	1	0.00	0.03	-0.03	-0.03
Mn (IV)	2	0.01	0.02	-0.01	-0.01
SO_4	8	0.16	0.02	0.13	-1.07
O_2	2	0.02	0.01	0.01	-0.03
Electrons transferred from OC mineralization					
DIC	-4	2.56	3.72	-1.16	4.66

Table S5. Calculated number of electrons transferred during organic carbon oxidation across flow path from DP6b to river.

Electron Donors (ED)	Electrons Transferred Per Reaction (mol)	DP6b (mmol)	River (mmol)	DP6b - river (mmol)	Electrons Transferred (meq/L)
DOC	-4	0.03	0.05	-0.02	0.09
Electron Acceptors (EA)					
Fe (III)	1	0.03	0.00	0.03	-0.03
Mn (IV)	2	0.02	0.00	0.02	-0.03
SO_4	8	0.02	0.09	-0.06	-0.51
O_2	2	0.01	0.03	-0.02	-0.04
Electrons transferred from OC mineralization					
DIC	-4	2.10	0.30	1.80	7.21

Table S6. Calculated number of electrons transferred during organic carbon oxidation across flow path from DP1c to DP7c.

Electron Donors (ED)	Electrons Transferred Per Reaction (mol)	DP1c (mmol)	DP7c (mmol)	DP1c - DP7c (mmol)	Electrons Transferred (meq/L)
DOC	-4	0.02	0.07	-0.05	0.21
Electron Acceptors (EA)					
Fe (III)	1	0.00	0.00	0.00	0.00
Mn (IV)	2	0.01	0.01	0.01	-0.01
SO_4	8	0.11	0.00	0.10	-0.83
O_2	2	0.01	0.01	0.00	0.00
Electrons transferred from OC mineralization					
DIC	-4	1.51	2.10	-0.59	2.36

Table S7. The key chemical reactions involving Fe and As cycling in the HZ of the riverbanks under dynamic flow condition.

	Reaction name	Reaction equation
R1	SOC dissolution to DOC	SOC = DOC
R2	DOC respiration	CH_2O (DOC) + O_2 = CO_2 + H_2O
R3	abiotic Fe(II) oxidation	$4Fe^{2+} + O_2 + 6H_2O = 4FeOOH + 8H^+$
R4	Fe(III) oxide dissolution /DOC mineralization	$4FeOOH$ + CH_2O (DOC) +$8H_2O$ = $4Fe^{2+}$ + HCO_3^- (DIC) + $7H^+$ + $5OH^-$
R5	As adsorption	$H_3AsO_3 + FeOH = H_2O + FeH_2AsO_3$

Video S1: Tidal fluctuations in a riverbank aquifer during dry season along the Meghna River, Bangladesh. The green circles represent the hydraulic heads in piezometers (PZ wells) augured in with screen intervals that spanned the vertical range of tidally-driven water table fluctuations during dry season. Red circles represent the hydraulic heads in 5 m deep monitoring wells (MWa wells) and green circles represent the hydraulic heads in 20 m deep monitoring wells (MWc wells). Black triangle represents the river stage. The cause of the fluctuations is the semi-diurnal tides in the Bay of Bengal. https://youtu.be/EqlDiIHC6Lw.

REFERENCES

(68) Weinman, B.; Goodbred, S. L.; Zheng, Y.; Aziz, Z.; Steckler, M.; van Geen, A.; Singhvi, A. K.; Nagar, Y. C. Contributions of Floodplain Stratigraphy and Evolution to the Spatial Patterns of Groundwater Arsenic in Araihazar, Bangladesh. *Bull. Geol. Soc. Am.* **2008**, *120* (11–12), 1567–1580. https://doi.org/10.1130/B26209.1.

(69) Meng, X.; Korfiatis, G. P.; Jing, C.; Christodoulatos, C. Redox Transformations of Arsenic and Iron in Water Treatment Sludge during Aging and TCLP Extraction. *Environ. Sci. Technol.* **2001**, *35* (17), 3476–3481. https://doi.org/10.1021/es010645e.

(70) Hug, S. J.; Leupin, O. X.; Berg, M. Bangladesh and Vietnam: Different Groundwater Compositions Require Different Approaches to Arsenic Mitigation. *Environ. Sci. Technol.* **2008**, *42* (17), 6318–6323. https://doi.org/10.1021/es7028284.

(71) Bouwer, H. & Rice, R. C. A Slug Test for Determining Hyraulic Conductivity of Unconfied Aquifers with Completely or Partially Penetrating Wells. *Water Resour. Res.* **1976**, *12*, 423–428.

(72) Ferris, J. G. Cyclic Fluctuations of Water Level as a Basis for Determining Aquifer Transmissibility. *Int. Union Geod. Geophys.* **1951**, *2*, 148–155.

(73) Bufe, A.; Hovius, N.; Emberson, R.; Rugenstein, J. K. C.; Galy, A.; Hassenruck-Gudipati, H. J.; Chang, J. M. Co-Variation of Silicate, Carbonate and Sulfide Weathering Drives CO2 Release with Erosion. *Nat. Geosci.* **2021**, *14* (4), 211–216. https://doi.org/10.1038/s41561-021-00714-3.

(74) Majumder, R. K.; Halim, M. A.; Saha, B. B.; Ikawa, R.; Nakamura, T.; Kagabu, M.; Shimada, J. Groundwater Flow System in Bengal Delta, Bangladesh Revealed by Environmental Isotopes. *Environ. Earth Sci.* **2011**, *64* (5), 1343–1352. https://doi.org/10.1007/s12665-011-0959-2.

(75) Mihajlov, I. et al. Recharge of Low-Arsenic Aquifers Tapped by Community Wells in Araihazar, Bangladesh, Inferred from Environmental Isotopes. *Water Resour. Res.* **2016**, *52*, 3324–3349.

(76) Parnell, A. & Govan, E. *simmr: A Stable Isotope Mixing Model. R package version 0.5.0.9000*. **2023**, https://CRAN.R-project.org/package=simmr.

(77) Parnell, A. C.; Phillips, D. L.; Bearhop, S.; Semmens, B. X.; Ward, E. J.; Moore, J. W.; Jackson, A. L.; Grey, J.; Kelly, D. J.; Inger, R. Bayesian Stable Isotope Mixing Models. *Environmetrics* **2013**, *24* (6), 387–399. https://doi.org/10.1002/env.2221.

(78) Liu, J.; Han, G. Tracing Riverine Particulate Black Carbon Sources in Xijiang River Basin: Insight from Stable Isotopic Composition and Bayesian Mixing Model. *Water Res.* **2021**, *194*, 116932. https://doi.org/10.1016/j.watres.2021.116932.

(79) Hudak, P. F. Chloride/Bromide Ratios in Leachate Derived from Farm-Animal Waste. *Environ. Pollut.* **2003**, *121* (1), 23–25. https://doi.org/10.1016/S0269-7491(02)00211-7.

(80) Biester, H.; Selimović, D.; Hemmerich, S.; Petri, M. Halogens in Pore Water of Peat Bogs - The Role of Peat Decomposition and Dissolved Organic Matter. *Biogeosciences* **2006**, *3* (1), 53–64. https://doi.org/10.5194/bg-3-53-2006.

(81) Biester, H.; Hermanns, Y. M.; Martinez Cortizas, A. The Influence of Organic Matter Decay on the Distribution of Major and Trace Elements in Ombrotrophic Mires - a Case Study from the Harz Mountains. *Geochim. Cosmochim. Acta* **2012**, *84*, 126–136. https://doi.org/10.1016/j.gca.2012.01.003.

(82) Vengosh, A. & Pankratov, I. Chloride/Bromide and Chloride/Fluoride Ratios of Domestic

Sewage Effluents and Associated Contaminated Ground Water. *Ground Water* **1998**, *36*, 815–824.
(83) DPHE/BGS. *Arsenic Contamination of Groundwater in Bangladesh*; 2001.
